# Three feminizing *Wolbachia* strains in a single host species: comparative genomics paves the way for identifying sex reversal factors

**DOI:** 10.3389/fmicb.2024.1416057

**Published:** 2024-08-22

**Authors:** Pierre Grève, Bouziane Moumen, Didier Bouchon

**Affiliations:** Université de Poitiers, Ecologie et Biologie des Interactions, UMR CNRS 7267, Poitiers, France

**Keywords:** *Wolbachia*, feminization, *Armadillidium vulgare*, genomics, isopod crustacean, effectors, *f* element

## Abstract

**Introduction:**

Endosymbiotic bacteria in the genus *Wolbachia* have evolved numerous strategies for manipulating host reproduction in order to promote their own transmission. This includes the feminization of males into functional females, a well-studied phenotype in the isopod *Armadillidium vulgare*. Despite an early description of this phenotype in isopods and the development of an evolutionary model of host sex determination in the presence of *Wolbachia*, the underlying genetic mechanisms remain elusive.

**Methods:**

Here we present the first complete genomes of the three feminizing *Wolbachia* (*w*VulC, *w*VulP, and *w*VulM) known to date in *A. vulgare*. These genomes, belonging to *Wolbachia* B supergroup, contain a large number of mobile elements such as WO prophages with eukaryotic association modules. Taking advantage of these data and those of another *Wolbachia*-derived feminizing factor integrated into the host genome (*f* element), we used a comparative genomics approach to identify putative feminizing factors.

**Results:**

This strategy has enabled us to identify three prophage-associated genes secreted by the Type IV Secretion System: one ankyrin repeat domain-containing protein, one helix-turn-helix transcriptional regulator and one hypothetical protein. In addition, a latrotoxin-related protein, associated with phage relic genes, was shared by all three genomes and the *f* element.

**Conclusion:**

These putative feminization-inducing proteins shared canonical interaction features with eukaryotic proteins. These results pave the way for further research into the underlying functional interactions.

## Introduction

The central role of host-symbiont interactions in the biology, ecology and evolution of the biotic world is now well recognized, which has led to the popularization of the holobiont concept (McFall-Ngai et al., 2013; Bordenstein and Theis, 2015). There are therefore no macroorganisms that do not host symbionts. Among these symbionts is *Wolbachia pipientis* (hereafter *Wolbachia*), a bacterium originally described in the mosquito *Culex pipiens* (Hertig and Wolbach, 1924). Since then, many studies have shown that this maternally inherited intracellular symbiont, belonging to the order Rickettsiales of the Alphaproteobacteria, exhibited exceptional traits. It is the most widespread endosymbiont in the animal world, representing a very wide diversity of strains infecting ~50% of arthropods and several nematodes (Zug and Hammerstein, 2012; Kaur et al., 2021). This enormous genetic diversity is reflected in the classification of *Wolbachia* where at least 17 phylogenetic supergroups (named A-F, H-Q and S; Supergroups A and B being the most numerous) can be identified to date (Kaur et al., 2021). Furthermore, these bacteria, often referred to as reproductive parasites, have attracted attention for the diversity of phenotypes they induce in their terrestrial arthropod hosts. Indeed, one of the main characteristics of *Wolbachia* is its remarkable ability to interfere with the reproduction of its hosts, through four main phenotypes, to optimize its own transmission, which has earned it the title of master manipulator (Werren et al., 2008).

Cytoplasmic Incompatibility (CI) represents the most common *Wolbachia*-induced phenotype (Werren et al., 2008; Landmann, 2019), resulting in embryonic death in crosses between infected males and uninfected females. It has been observed in Insecta as well as in Acari and in Isopoda (Landmann, 2019). The molecular basis of CI has recently been demonstrated by the identification of two genes, *cifA* and *cifB*, from the Eukaryotic Association Module (EAM) of *Wolbachia*’s prophage WO (Shropshire and Bordenstein, 2019; Shropshire et al., 2020). The other three *Wolbachia*-induced phenotypes lead to sex ratio biases in the host. Male-killing (MK), first described in the ladybird *Adalia bipunctata,* results in males death favoring female survival (Hurst et al., 1999; Fukui et al., 2015). As for CI, a candidate MK gene (termed WO-mediated killing *wmk*) was identified in the EAM of WO prophage (Perlmutter et al., 2019). *Wolbachia*-induced parthenogenesis (PI) was first described in *Trichogramma* Hymenoptera (Stouthamer et al., 1990) in which infected females can produce daughters from unfertilized eggs (Ma and Schwander, 2017). Recently, two putative PI-inducing factors (PifA and PifB) has been identified, also localized in EAM (Fricke and Lindsey, 2024). *Wolbachia*-induced feminization, which consists in the feminization of genetic males, is a reproductive manipulation that has been widely described in isopods and also observed in two insect species (Hiroki et al., 2002; Negri et al., 2006; Bouchon et al., 2008). However, the mechanisms involved are different: in butterflies, the feminizing *Wolbachia* interact with the master regulator genes that control sex determination, whereas in crustaceans, they interact with the hormonal regulatory genes (Kageyama et al., 2017; Herran et al., 2021).

The first description of feminization in isopods was due to the pioneering work on the pill bug *Armadillidium vulgare* (Juchault et al., 1974). The identification of *Wolbachia* (*w*VulC strain) as the feminizing factor came later (Rousset et al., 1992; Bouchon et al., 1998). Two additional *Wolbachia* (*w*VulM and *w*VulP strains), have been identified in *A. vulgare* (Cordaux et al., 2004; Verne et al., 2007). In *A. vulgare*, genetically (ZZ) male embryos carrying *Wolbachia* inherited from the mother develop into functional females morphologically undistinguishable from genetic females (ZW). Several studies have suggested that feminization of genetic males results from inhibition of androgenic gland differentiation that produce the androgenic hormone [review in Bouchon et al. (2008) and Herran et al. (2020)]. As males inverted in females produce female-biased broods, all *Wolbachia*-infected females were ZZ individuals in natural populations (Juchault et al., 1993). This situation has given rise to strong genetic conflicts with major evolutionary consequences for interactions between *Wolbachia* and *A. vulgare* (Rigaud, 1997). In particular, it has been suggested that a feminizing factor called *f* element, derived from the *w*VulC *Wolbachia* strain, was at the origin of a new W-type chromosome (Juchault and Mocquard, 1993). This hypothesis has recently been verified, as the *f* element corresponds to the insertion of a large part of the *Wolbachia w*VulC chromosome into the *A. vulgare* genome (Leclercq et al., 2016). It has also been shown that *w*VulC and the *f* element never co-occur (Durand et al., 2023).

Until now, genomic data on *Wolbachia* endosymbionts from woodlice has been very fragmentary, with only two genome assemblies. One is *w*Con, known to induce CI in *Cylisticus convexus* (Moret et al., 2001; Badawi et al., 2018). The other one available in databases is an assembly of 10 contigs from the genome of *w*VulC. In this study, we performed a comparative genomic analysis of the three *Wolbachia* strains identified so far in *A. vulgare*. We obtained the complete genomes of *w*VulC, *w*VulM using both short and long read sequencing and *w*VulP strains using long read sequencing. Our data shed light on the evolution of *Wolbachia* strains in *A. vulgare* and highlighted putative candidate feminization genes.

## Materials and methods

### Host lineages and DNA extraction

Four *A. vulgare* lineages were used in this study: a *Wolbachia*-free lineage (called BF) collected in 1967 in Nice (France), a *w*VulC-infected lineage (called ZN) collected in 1991 in Celles-sur-Belle (France), a *w*VulM-infected lineage (called BI) collected in 1999 in Méry sur Cher (France) and a *w*VulP-infected lineage (called CP) collected in 2007 in Poitiers (France) (Cordaux et al., 2004; Verne et al., 2007). These lineages have since been stably maintained in the laboratory, at 20°C under natural photoperiod, with food *ad libitum* (dead lime leaves and carrots). Controlled rearing on a standard diet homogenizes the diversity of the gut microbiota and no other sex-parasitic bacteria have been identified (Dittmer et al., 2014; Dittmer and Bouchon, 2018).

The sex ratios observed in the lineages were recorded each year. The *Wolbachia*-free BF lineage is used as a control for genetic sex determination as attempts to cure individuals with antibiotics have been unsuccessful (Rigaud and Juchault, 1998). The proportion of males was determined in each brood over 5 years and visualized by boxplots using arcsine transformations. Comparison of the mean male ratio was performed in R version 4.3.2 (R Core Team, 2023) through a generalized linear mixed model with host lineage as fixed effect and clutch size and year as random effects. Assessment of the model was performed using the *performance* package (Lüdecke et al., 2021).

DNA extraction was carried out by homogenizing ovaries of 30 to 50 infected females with a Dounce tissue grinder B in a PBS solution supplemented with sucrose (0.25 M) and L-glutamine (5 mM) which allow the cells to be crushed but not the nuclei. Large fragments were removed by passing the solution through a 5 μm filter. The remaining nuclei were pelleted after centrifugation at 200 x g (4°C, 20 min.). The supernatant was then centrifuged at 4100 x g to pellet the bacteria (4°C, 20 min.). DNA purification was performed using the Qiagen DNeasy Blood and Tissue Kit as follows. The *Wolbachia*-enriched pellet was first resuspended in 180 μL ATL buffer plus 20 μL of proteinase K (10 mg/mL) and incubated for 1 h at 54°C. After treatment with RNase A (0.2 μg/μL, at 37°C for 15 min.), DNA was recovered following the manufacturer’s instructions. DNA quantification was performed using a Nanodrop 1000 spectrophotometer (Thermo Scientific) and the Qubit 2.0 fluorimeter (Invitrogen).

### Genome sequencing, assembly, and annotation

Library preparation for nanopore sequencing was performed using the protocols for the SQK-LSK109 Ligation Sequencing Kit (Oxford Nanopore Technologies, UK). Libraries were sequenced on R9.4.1 flowcells on a MinION sequencer for 48 h. Base-calling was performed using the Guppy base-caller software v4.2.2 (Oxford Nanopore Technologies, UK) using high-accuracy mode, with a quality score cut-off of 9 and minimum read length filter set of 200. Adapters were trimmed with Porechop 0.2.4^1^ on the basecalled reads.

Prior to Illumina sequencing, targeted genome enrichment was used for *w*VulC and *w*VulM strains as described in Geniez et al. (2012). Illumina libraries were prepared and samples were sequenced at HudsonAlpha Genome Sequencing Center (Hunstville AL35806, USA) on the Illumina HiSeq 2000 (Geniez, 2013).

These two sequencing strategies led to 2,001,609 and 2,581,285 Nanopore long reads and 72,894,720 and 64,625,816 Illumina reads for *w*VulC and *w*VulM, respectively. The *w*VulP genome was assembled from 1,795,327 Nanopore long reads.

Bacterial genome assembly was performed using the long reads assembler Flye v2.8.1 (Kolmogorov et al., 2019). The best assembly was chosen based on the expected size and circular status of the genome, after comparing meta and single mode assembly using several overlap parameters. The resulting assemblies were first polished with Nanopore reads using Nanopolish v0.14.0 (Loman et al., 2015). Additional Illumina polishing using Medaka v1.6.0 was performed on assemblies for *w*VulC and *w*VulM strains after assessing the quality of Illumina reads with FastQC v0.11.9 (Andrews, 2010) and removing adapters and quality filtering using Fastp v0.21.0 (Chen et al., 2018). Genome completeness was assessed by using the Benchmarking Universal Single-Copy Orthologs (BUSCO) pipeline v5.4.6 and the rickettsiales_odb10 database (Manni et al., 2021). The polished assemblies with the highest BUSCO score were selected for further analysis.

The genomes were functionally annotated using the NCBI Prokaryotic Genome Annotation Pipeline (PGAP) (Tatusova et al., 2016).

### Comparative genomics and phylogenomics

The three *Wolbachia* genomes were presented starting with *dnaA* gene as for wMel (Wu et al., 2004) and most other *Wolbachia* genomes. Comparisons between the three genomes were performed and visualized using FastANI v1.3.3 (Jain et al., 2018) implemented in the NanoGalaxy platform (de Koning et al., 2020) and Mauve v2.4.0 progressive alignments (Darling et al., 2010) using Circos software v0.69–9 (Krzywinski et al., 2009).

Comparisons of *w*VulC genome with a previous draft genome (GCA_001027565.1) and the *w*VulC inserts identified into the pill bug nuclear genome (Leclercq et al., 2016) were performed using FastANI v1.3.3 (Jain et al., 2018) implemented in the NanoGalaxy platform (de Koning et al., 2020).

Orthofinder v2.5.5 (Emms and Kelly, 2019) implemented in the NanoGalaxy platform (de Koning et al., 2020) was used to identify orthologous sequences and infer the species tree from 29 *Wolbachia* genomes belonging to the six A-F supergroups. Four hundred and fourteen single-copy proteins were aligned with MAFFT v. 7.505 (Katoh et al., 2017). The concatenated alignment was used for phylogenetic reconstruction by maximum-likelihood with the iQtree 2.1.2 (Minh et al., 2020) implemented in the NanoGalaxy platform (de Koning et al., 2020). The best model (JTT + F + R6) was selected by ModelFinder (Kalyaanamoorthy et al., 2017), implemented in IQ-TREE, based on Bayesian Information Criterion. Branch support was assessed using ultrafast bootstrap with 1,000 replicates. The resulting consensus tree was drawn using iTol v6.8.2 (Letunic and Bork, 2021).

### Analyses of *Wolbachia* prophage regions, mobile elements, T4SS, effectors, and biotin operon

Prophage regions (WO prophages) in the three *Wolbachia* assemblies were estimated using the PHASTEST web server (Wishart et al., 2023). WO phage genomic maps were drawn using the *gggenes* package v.0.5.1 in R (Wilkins, 2023). EAMs (Bordenstein and Bordenstein, 2016; Bordenstein and Bordenstein, 2022) were identified by annotating CDSs flanking or located within the PHASTEST-predicted prophage regions through BLASTp queries using the NCBI clustered protein database. Alignment of the large serine recombinase of the WO prophages was generated with MAFFT v. 7.505 (Katoh et al., 2017) and the phylogeny was inferred by maximum likelihood using the iQtree server v1.6.12 (Trifinopoulos et al., 2016). The best substitution model (JTT + F + G4) was selected by ModelFinder (Kalyaanamoorthy et al., 2017) and tree topology was tested by ultrafast bootstrap (Minh et al., 2013) of 1,000 iterations.

Insertion sequence (IS) elements were determined in the three genomes using the ISEScan v1.7.2.3 (Xie and Tang, 2017) implemented in the NanoGalaxy platform (de Koning et al., 2020). Candidate intron sequences were identified using RASTtk pipeline (Brettin et al., 2015) and BLASTp searches against the Database for Bacterial Group II Introns (Candales et al., 2012). The comparative location of mobile elements between the three genomes was represented using Circos software v0.69–9 (Krzywinski et al., 2009).

Prediction of intact secretion systems and secreted proteins was performed using EffectiveDB queries v5.2 (Eichinger et al., 2016).

Genes of the biotin operon were identified by BLASTn using the sequences previously identified in the *w*VulC draft genome. Comparison of the structure of the operons in the three *Wolbachia* genomes was visualized using *gggenes* R package (Wilkins, 2023).

## Results

### Sex ratio bias in *Wolbachia*-infected host lineages

**Figure 1:**
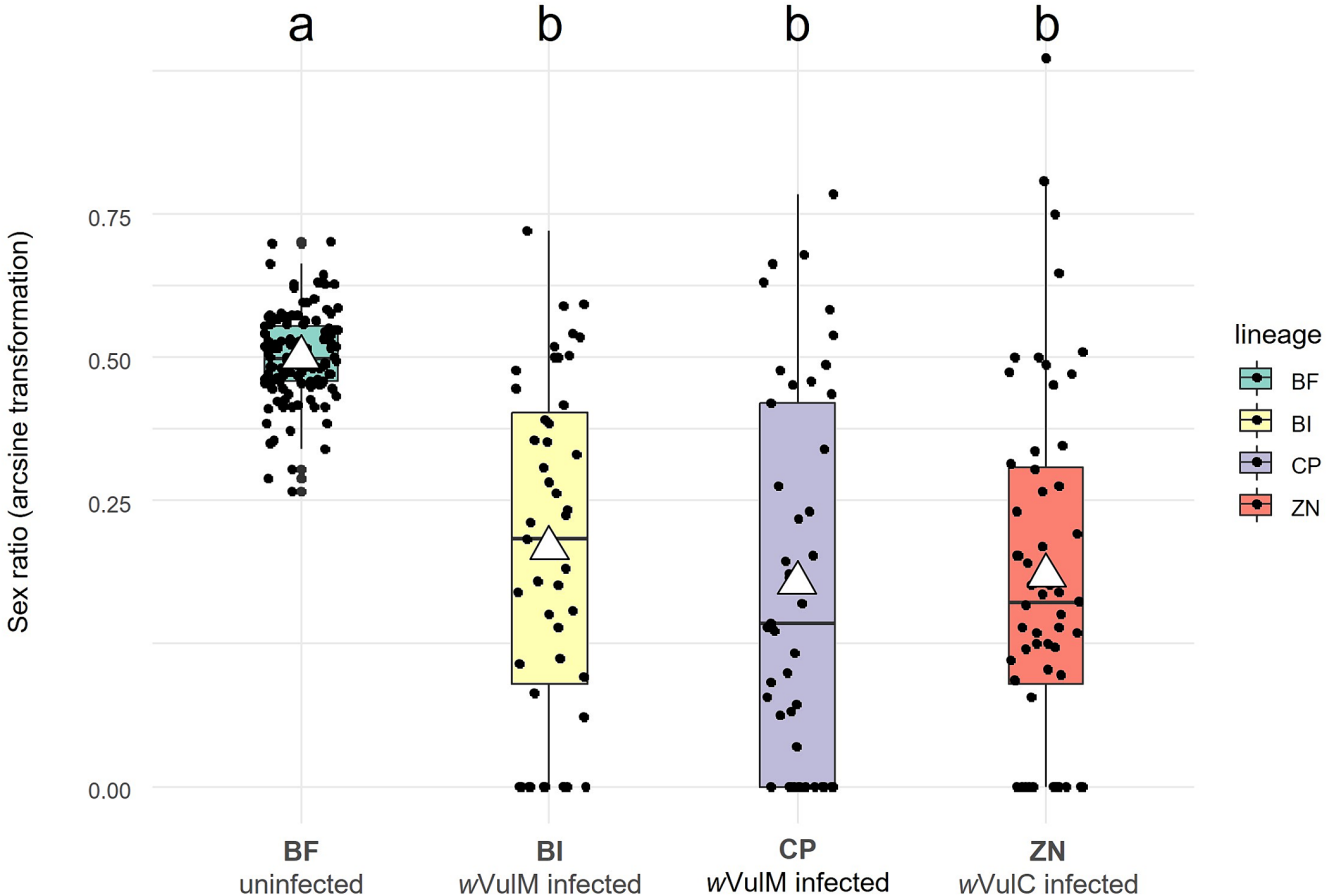
Boxplot showing sex ratios (proportion of males) of the four laboratory lineages of *A. vulgare* (BF, BI, CP, and ZN) over five years. BF lineage is uninfected whereas BI, CP, and ZN lineages are infected with *w*VulM, *w*VulP, and *w*VulC *Wolbachia* strains, respectively. Sex ratios have been arcsine transformed. White triangles correspond to mean values. Statistical analysis was performed using a Generalized Linear Mixed Model (conditional R-squared = 0.222).

The *Wolbachia* strains sequenced in this study have been isolated from females belonging to three different lineages of *A. vulgare,* which exhibit sex ratio biases (Figure 1; Supplementary Table S1; Supplementary Figure S1). The male proportions (mean ± se) were 19.71 ± 0.03% for ZN lineage (infected with *w*VulC), 23.45 ± 0.03% for BI lineage (infected with *w*VulM), and 19.96 ± 0.03% for CP lineage (infected with *w*VulP) with no significant differences. The greater variance in male proportions in the infected lineages reflected variations in the *Wolbachia* transmission rate (Figure 1). In contrast, the uninfected BF control line showed a balanced sex ratio of close to 50% (49.9 ± 0.01%).

### Comparative genomics of the three feminizing strains

**Figure 2:**
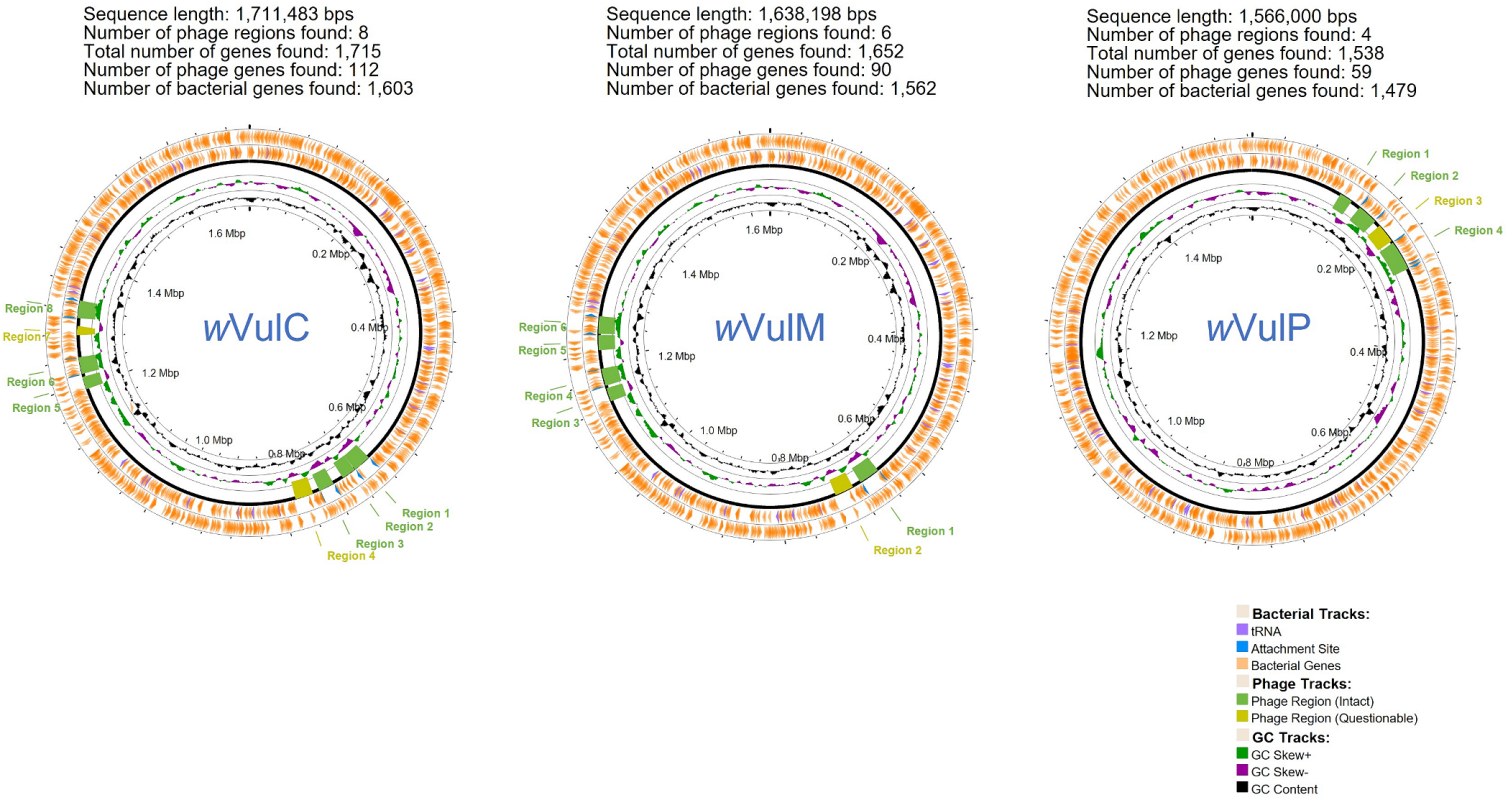
Graphical representation of the circular genome map of the three *Wolbachia* strains *w*VulC, *w*VulM, *w*VulP. PHASTEST description, from the outside to the center: (1) Predicted CDSs transcribed in the counterclockwise direction. (2) Predicted CDSs transcribed in the clockwise direction. (3) Predicted phage regions (intact and questionable). (4) GC skew. (5) GC content.

**Figure 3:**
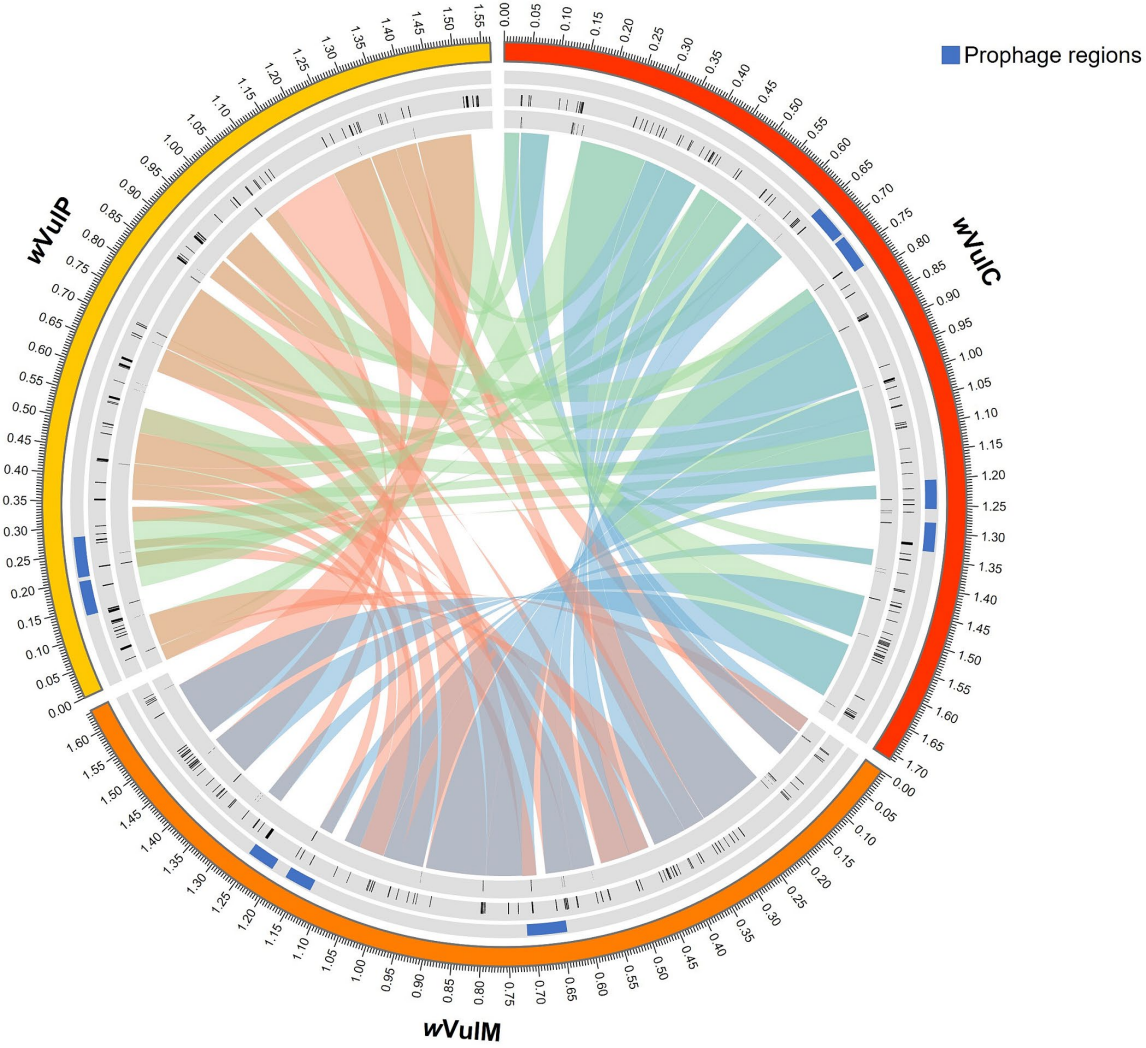
Circos representation of the progressive Mauve alignment and mobilome of the three *Wolbachia* strains (*w*VulC, *w*VulM, *w*VulP) of *A. vulgare*. First inner ring: WO prophage regions in blue. Second inner ring: complete IS elements in black. Third inner ring: group II introns in black.

The three *Wolbachia* genomic sequences were assembled into single molecules of 1,711,483 bp for the *w*VulC strain, 1,638,198 bp for the *w*VulM strain and 1,566,000 bp for the *w*VulP strain, with a G + C content ranging from 34.8 to 34.9% (Figure 2). The two *w*VulC and *w*VulM genomes were circularized and even though *w*VulP was not completely closed, it was a genome of the same quality assembled into a single scaffold. The general features of the genomes are presented in Table 1. Their sizes were comparable to those of other *Wolbachia* strains inducing reproductive phenotypes (Sun et al., 2001), with each genome containing between 1,282 and 1,484 protein-coding genes (Table 1). The *w*VulC, *w*VulM, and *w*VulP assemblies were assessed by BUSCO, showing a very small number of fragmented and missing genes (Table 1), resulting in high scores (99.8, 99.5 and 98.3% for *w*VulC, *w*VulM, and *w*VulP, respectively). The three genomes were very similar as shown by the high ANI value obtained from pairwise comparisons (> 98%, Supplementary Table S2). However, numerous genomic rearrangements have been identified, particularly in the *w*VulP genome (Figures 2, 3; Supplementary Figures S2A–C). Orthofinder analysis of the three genomes revealed 1,094 orthogroups in common out of 1,259 (Supplementary Table S3). *w*VulC and *w*VulM shared 126 orthogroups whereas each of these strains shared only 15 and 10 orthogroups with *w*VulP. Finally, one to 11 specific orthogoups corresponded to four, three and twenty-four specific genes of *w*VulC, *w*VulM, and *w*VulP, respectively (Supplementary Table S3). The *w*VulC specific genes were two hypothetical protein paralogs (wVulc_000316 and wVulc_000382; 100% amino acid identity) and two DUF4815 domain-containing protein paralogs (wVulc_000708 and wVulc_001210; 99.7% amino acid identity). In *w*VulM, the three specific genes were paralogs of recombinase (wVulm_000691, wVulm_001174 and wVulm_001253; 100% amino acid identity) corresponding to the third recombinase in the core module prophage WOVulM1_2, WOVulM3_4, and WOvulM5_6 (see below). As to the *w*VulP-specific genes, five were duplicated mobile elements (IS and group II introns), one was a duplicated phage tail protein, one was a duplicated SET domain-containing protein, one was a duplicated ANK gene and three were duplicated hypothetical proteins. Each duplicated gene was 100% identical with the exception of the phage tail paralogs which were 77% identical (Supplementary Table S3).

**Table 1:** Comparative statistics and BUSCO assessment of the three *Wolbachia* genomes *w*VulC, *w*VulM, and *w*VulP isolated from *A. vulgare*.

	Strain		
	*w*VulC	*w*VulM	*w*VulP
Length (bp)	1,711,483	1,638,198	1,566,000
%GC	34.91	34.84	34.85
Genes	1,708	1,649	1,535
CDS	1,666	1,607	1,494
Protein-coding genes	1,448	1,386	1,282
Pseudogenes	218	221	212
rRNA	3	3	3
tRNA	35	35	34
ncRNA	3	3	3
tmRNA	1	1	1
BUSCO assessment			
Complete and Single	362	361	356
Duplicated	1	1	2
Fragmented	0	1	4
Missing	1	1	2

Furthermore, comparison of the complete, closed *w*VulC genome with a previous draft genome obtained by Sanger sequencing showed a high ANI value of 99.9%. The non-collinearity between these two sequences was due to artificial joining of the 10 contigs in the Sanger draft assembly (Supplementary Figure S3). Comparison of the *w*VulC genome was also carried out with the *f* element (derived from *w*VulC) which comprised nine scaffolds spanning 3.13 Mb (Leclercq et al., 2016). This showed a high average nucleotide identity of 99.6% (Supplementary Figure S4) but revealed numerous genomic rearrangements indicating multiple insertion and duplication events of the *f* element in the *A. vulgare* genome.

### Phylogenomic relationships

**Figure 4:**
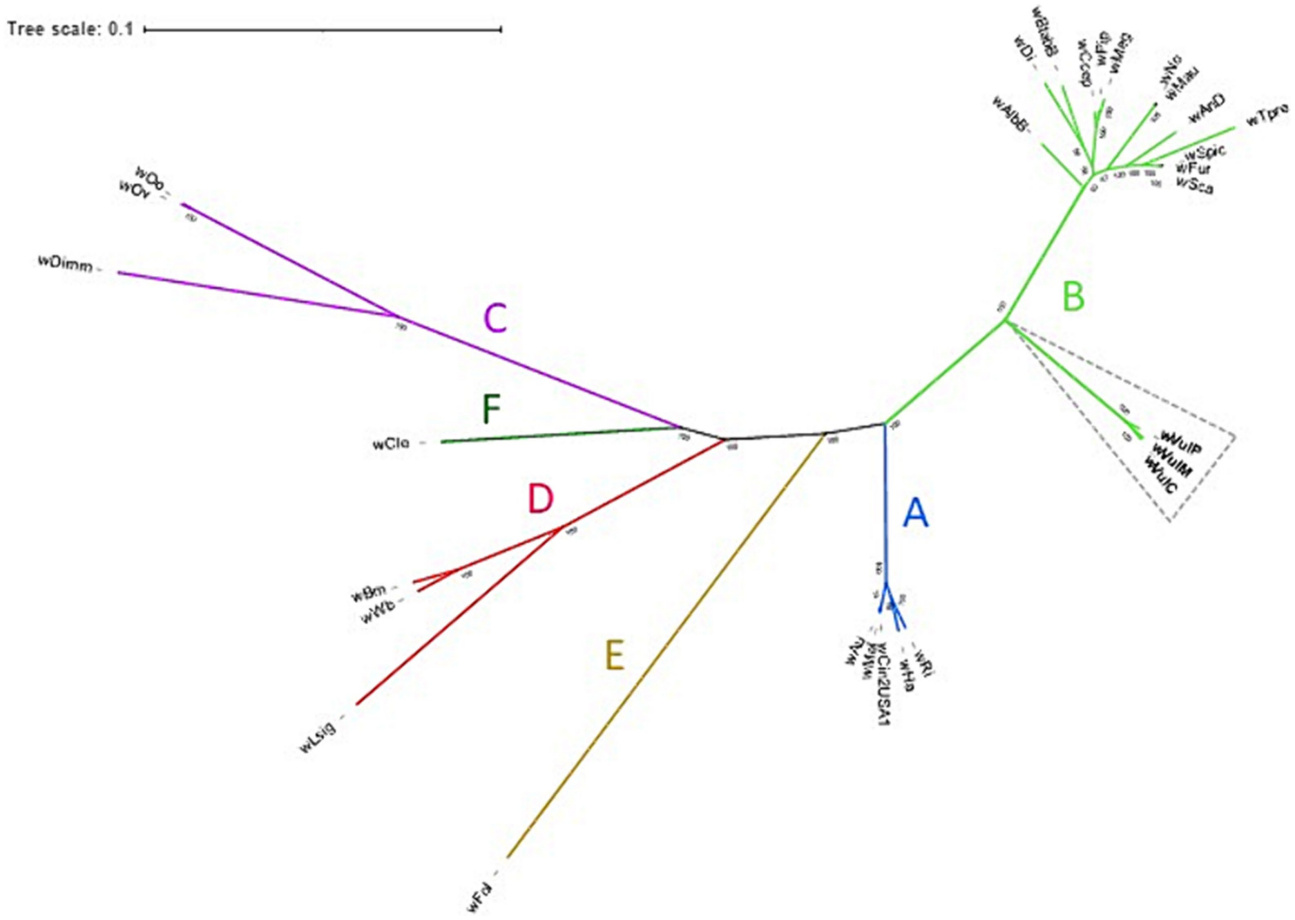
Unrooted maximum-likelihood consensus tree based on concatenated alignments of 415 single-copy orthologues in 29 *Wolbachia* genomes. Newly assembled genomes are shown in bold. Tree scale bar represents substitutions per site. Bootstrap support values (> 50%) from 1,000 replicates are shown at the edges.

The phylogenomic position of the three *Wolbachia* from *A. vulgare* was determined by comparison with 26 publicly available and annotated *Wolbachia* genomes, including five from supergroup A, thirteen from supergroup B, three from supergroup C, three from supergroup D and one for supergroup E and F strains from various host species (Supplementary Table S4). A total of 415 single-copy gene ortholog clusters were used to reconstruct the phylogenomic tree (Figure 4; Supplementary Table S5). The three *Wolbachia* strains isolated from *A. vulgare* belonged to the supergroup B, forming a separated clade, with *w*VulC and *w*VulM being closely related (bootstrap support 100, Figure 4).

### Mobilome identification

ISEScan predicted 105, 103 and 77 complete IS elements from 10 different families in *w*VulC, *w*VulM and *w*VulP, respectively, representing around 8% of the genome size (Supplementary Table S6). The IS families most represented in the three genomes, when complete sequences were taken into account, were IS110, IS256, IS3, IS4, IS5 with the exception of *w*Vulp where IS3 had only one representative. Specific IS clusters were ISNCY_191 for *w*VulC, IS110_236, new_343 and IS4_64 for *w*VulP (Supplementary Table S6). Many IS element positions coincided with regions where synteny breaks occurred (Figure 3), suggesting that IS elements may have contributed to the genomic rearrangements among the three genomes.

RASTtk annotations and BLASTp searches against the Database for Bacterial Group II Introns showed 29, 25 and 29 group II intron-associated genes in *w*VulC, *w*VulM and *w*VulP, respectively (Supplementary Table S7). The sum of the lengths of these genes was 17,892 bp, 14,556 bp, and 13,332 bp respectively, representing 0.9–1% of the genome size. The distribution of these introns was not homogeneous and regions of several successive genes were observed (Figure 3). As with IS elements, these group II introns are often located in regions of synteny breaks (Figure 3).

Prophage regions were identified by PHASTEST and BLASTp searches using annotated CDS, both in predicted and flanking regions. The results were also manually curated using the recently published WO prophage annotations (Bordenstein and Bordenstein, 2022). This strategy enabled us to extend the regions initially predicted by PHASTEST (Figure 2), notably by including EAMs (Bordenstein and Bordenstein, 2016). Functional annotations of these regions were shown in Supplementary Table S8.

**Figure 5:**
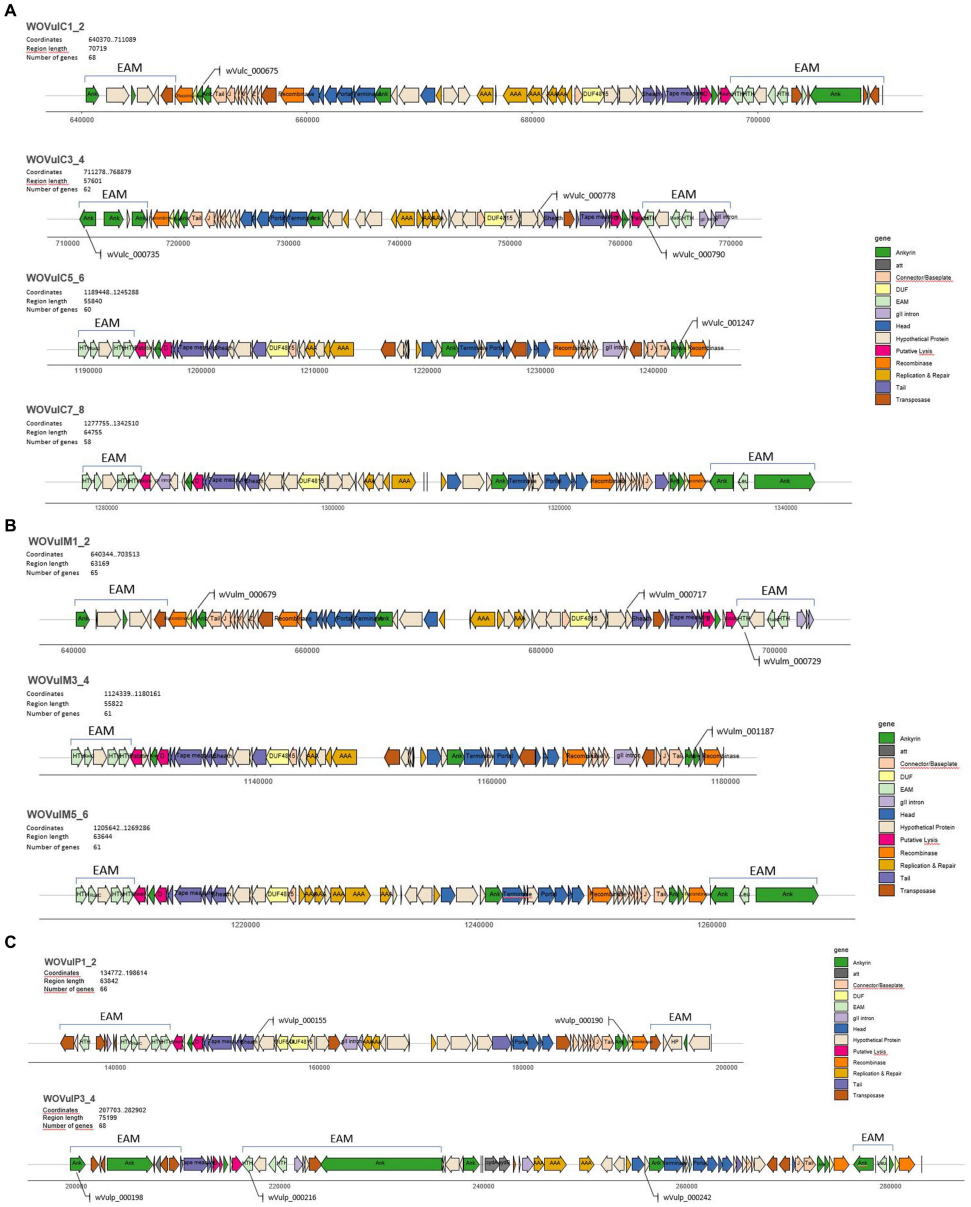
Annotation of prophage regions of *w*VulC, *w*VulM, *w*VulP by PHASTEST and manual curation. **(A)** *w*VulC harbors four intact prophage regions (70.7 kb, 57.6 kb, 55.8 kb and 64.8 kb). **(B)** *w*VulM harbors three intact prophage regions (63.2 kb, 55.8 kb, and 63.6 kb) and **(C)** *w*VulP harbors two intact prophage regions (63.8 kb and 75.2 kb). Lengths of the graphs are proportional to the size (bp) of the prophages. The legends indicate the color coding of the modules. EAM-like regions are highlighted. Locus tags of T4SS effectors are indicated.

We identified four complete prophage regions in *w*VulC measuring 70,719 bp (WOVulC1_2), 57,601 bp (WOVulC3_4), 55,840 bp (WOVulC5_6), and 64,755 bp (WOVulC7_8). WOVulC1_2 and WOVulC3_4 formed a continuous region with two sets of all core prophage modules separated by two EAMs (Figure 5A; Supplementary Table S8). Three complete prophage regions were identified in *w*VulM measuring 63,169 bp (WOVulM1_2), 55,822 bp (WOVulM3_4) and 63,644 bp (WOVulM5_6) (Figure 5B; Supplementary Table S8). In *w*VulP, only two prophage regions of 63,842 bp (WOVulP1_2) and 75,199 bp (WOVulP3_4) were found (Figure 5C; Supplementary Table S8). They formed a continuous prophage region separated by two EAMs. In addition, WOVulP3_4 is interrupted by an internal EAM. Overall, these prophages accounted for around 10% of each genome. The core genomic content of each prophage WO began with a large serine recombinase and ended with a patatin. With the exception of prophages WOVulC3_4 and WOVulP1_2, the majority of prophages contained two different serine recombinase genes, the second downstream of the first. In tandem to the latter, a third short recombinase was specifically present in all three prophage regions of *w*VulM (Figure 5B; Supplementary Table S8).

**Figure 6:**
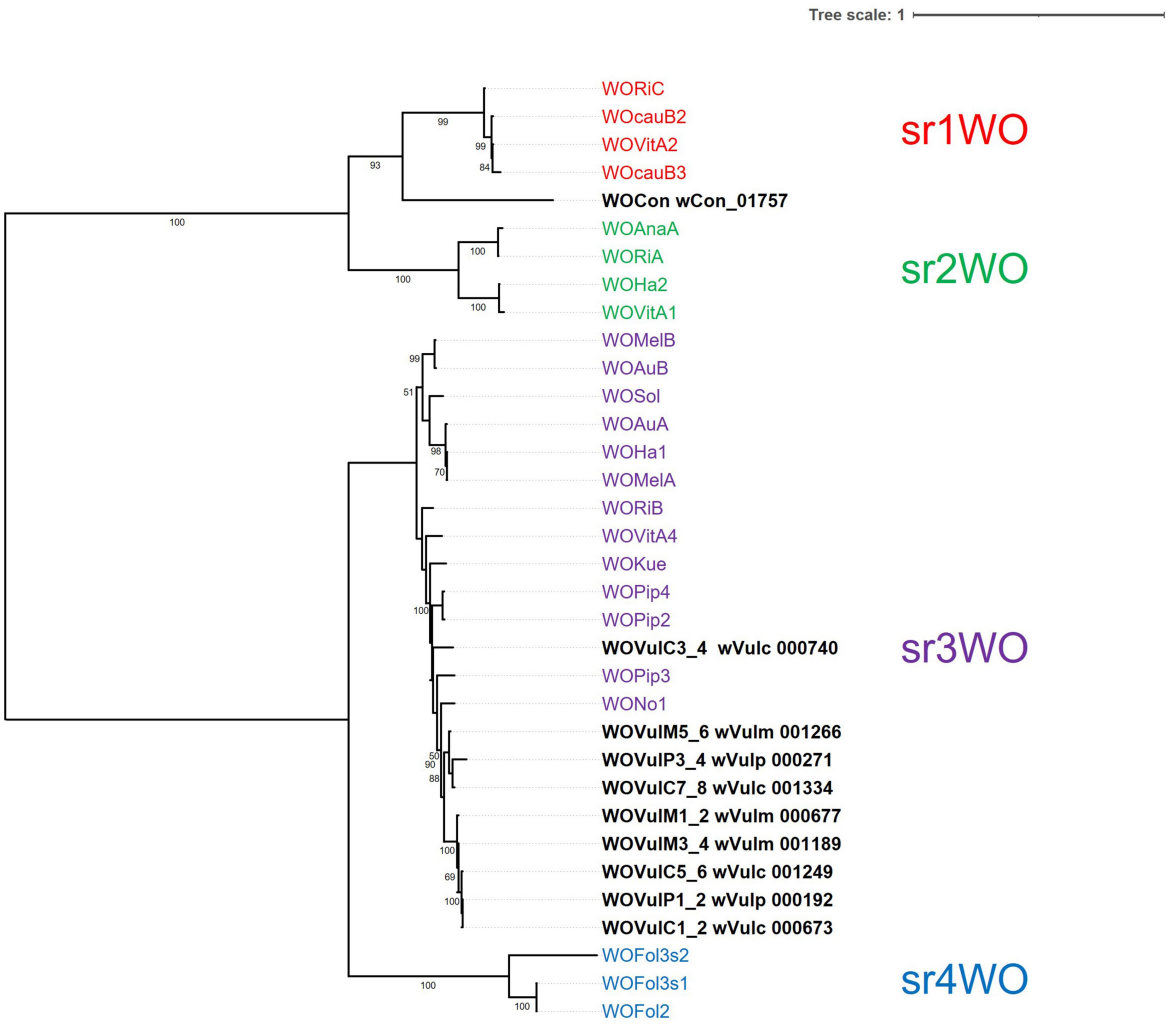
Maximum-likelihood phylogeny based on the large serine recombinase of *Wolbachia* prophage regions. The phylogeny was generated from 34 sequences (630 amino acid sites) using the JTT + F + G4 model. The tree was rooted at the mid-point. Tree scale bar represents substitutions per site.

Four distinct WO variants (sr1WO-sr4WO) have been described based on the large serine recombinase phylogeny and core module synteny (Bordenstein and Bordenstein, 2022). Based on the phylogeny of the first serine recombinase genes marking the start of the core modules, classification of the WO prophages of the three *Wolbachia* strains showed that they all belonged to the sr3WO group (Figure 6). The general structure of these prophages corresponded to the module synteny described for sr3WO variants, which includes an internal core prophage WO region flanked by EAM genes (Bordenstein and Bordenstein, 2022). Although the synteny of genes within each core module was consistent with the sr3WO organization (i.e., connector/baseplate, head, replication & repair, tail), variations between prophages were observed, some due to the presence of mobile elements (transposases and group II introns). These variations were consistent with the phylogenetic position established with the serine recombinase gene, with the closest prophages sharing a better synteny. In particular, strong collinearity is observed between the closely related core prophage modules WOVulC1_2 and WOVulM1_2, WOVulC5_6 and WOVulM_3_4 and WOVulC7_8 and WOVulM5_6 (Figure 6). In contrast, WOVulP3_4 had a particular structure with 3 EAM-like regions, including one region localized within the core prophage module (Figure 5C).

Using PHASTEST, we also examined the wCon assembly of 237 contigs and identified one large serine recombinase (locus wCon_01757) in an incomplete prophage region due to the end of the contig. This sequence was added in the phylogenetic tree, showing a putative prophage belonging to sr1WO cluster (Figure 6). This could reflect different prophage features depending on the *Wolbachia* reproductive phenotype.

Genes encoding proteins containing an ankyrin repeat domain (ANK genes) were localized in each core prophage module with a highly conserved arrangement in the three *Wolbachia* genomes (Figures 5A–C). At the beginning of the module, two contiguous ANK genes were located just after a DUF2924 domain-containing protein downstream of the large serine recombinase, then toward the middle, one ANK located just after the phage terminase large subunit gene (except for WOVulP1_2) and at the end of the module, one ANK gene located between a patatin-like phospholipase and a holin-like protein constituting a lytic cassette (Bordenstein and Bordenstein, 2022).

Regions similar to EAMs previously reported in other *Wolbachia* genomes were also observed in *w*VulC, *w*VulM and *w*VulP prophage regions (Figures 5A–C). These EAM-like regions contained genes encoding transcription regulators (helix-turn-helix HTH domain-containing protein) and ankyrin repeat proteins that might be involved in the host manipulation (Bordenstein and Bordenstein, 2022). *RadC* genes encoding JAB domain-containing proteins were also identified in EAMs from WOVulC1_2, WOVulC3_4, WOVulM1_2, and WOVulP1_2 prophages. Finally, mobile elements (Group II introns and transposases) were also frequent in EAMs. Here again, there was a strong collinearity between the EAMs of closely related prophages, with most EAMs being very similar in composition and structure, with the exception of those of WOVulP3_4.

### T4SS and phage-related putative feminizing effectors

The two operons characteristic of the T4SS were identified in the three genomes (Supplementary Table S9). The *virB3*-*virB6* operon was constituted of *virB3*, *virB4* and four *virB6* genes. The *virB8-virD4* operon was constituted of *virB8*, *virB9*, *virB10*, *virB11* and *virD4* genes. All these genes were highly conserved between the three genomes (96 to 100% of amino acid identity) with the exception of the second duplicated virB6 (83% amino acid identity).

The prediction of secreted proteins showed a high number of potential T4SS effectors among the total number of effectors: 97 (92 unique genes) out of 1,530 for *w*VulC, 100 (96 unique genes) out of 1,474 for *w*VulM and 83 (82 unique genes) out of 1,353 for *w*VulP (Supplementary Table S9). Among these T4SS effectors, particular attention was paid to genes present in the prophage regions and common to all three genomes and the *f* element. This led to the identification of genes encoding an ankyrin-repeat protein, an HTH-domain protein and a hypothetical protein.

Ten ANK genes were shared by all the three *Wolbachia* strains, including one which is localized in the core prophage modules. It is represented by two homologs (wVulc_000675 and wVulc_001247) in WOVulC1_2 and WOVulC5_6, two homologs (wVulm_000679 and wVulm_001187) in WOVulM1_2 and WOVulM3_4, and one homolog (wVulp_000190) in WOVulP1_2 (Figures 5A–C). These five ANK genes were highly conserved, from 98.9 to 100% amino-acid identity (Supplementary Table S9). Moreover, seven homologs of this gene were also present in the *f* insert (Wxf_00764, Wxf_00854, Wxf_01743, Wxf_02351, Wxf_02903, Wxf_0307 and, Wxf_03107) with an amino acid identity ranging from 77.2 to 97.4% (Supplementary Table S9).

A gene encoding a transcriptional regulator (HTH domain protein) shared by all three strains and the *f* element, was also predicted as a T4SS effector (Supplementary Table S9). This gene was localized in the EAMs of the WOVulC3_4 (wVulc_000790), WOVulM1_2 (wVulm_000729) and WOVulP3_4 (wVulp_000216) prophages (Figures 5A–C). Interestingly, five homologs (Wxf_00824, Wxf_00827, Wxf_00907, Wxf_01690 and Wxf_02404) of this gene were annotated in the largest scaffold of the *f* element. Conservation of residues of these sequences was low (around 37% of amino acid identity) but the HTH domain is still present.

Finally, 18 hypothetical proteins common to all three genomes were also predicted as T4SS effectors. Among them, one with 100% amino acid identity, was located in the WOVulC3_4, WOVulM1_2, and WOVulP1_2 (wVulc_000778, wVulm_000717 and wVulp_000155 respectively) (Supplementary Table S8; Figures 5A–C). This hypothetical protein was also identified in the *f* element, with a degree of conservation of around 70% (Supplementary Table S8; Wxf_00810, Wxf_00893, Wxf_01704, and Wxf_02390).

Another T4SS effector shared by the three genomes, a latrotoxin-related protein (wVulc_001131, wVulm_001070, and wVulp_000491) might be involved in host-*Wolbachia* interactions. This gene present a latrotoxin C-terminal domain characteristic of the latrotoxins of the black widow spider (Grishin, 1998). It was not located in the prophages but in a region containing phage relic, just two CDS upstream a phage terminase large subunit encoding gene. Interestingly, one homolog (Wxf_02470; 90.4% amino acid identity) was also present in the *f* element (Supplementary Table S9).

### Conservation of the biotin pathway

**Figure 7:**
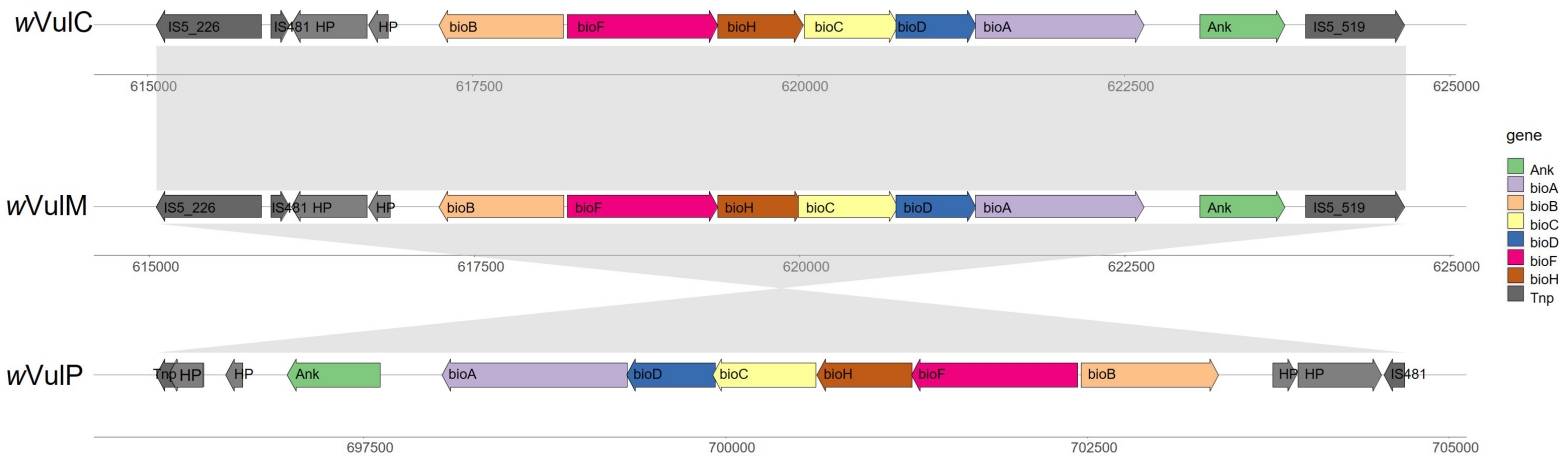
Comparison of the biotin operon of *w*VulC, *w*VulM, and *w*VulP and flanking regions showing collinearity.

A highly conserved biotin operon was identified in the three *Wolbachia* genomes (Figure 7), suggesting a potential supply of this vitamin by the bacteria, although this role is probably not essential given the non-obligatory nature of the association. This operon contained the six canonical biotin genes [*bioB*, *bioF*, *bioH*, *bioC*, *bioD* and *bioA;* (Gerth and Bleidorn, 2016)] which are highly conserved (99 to 100% amino acid sequence identity). This operon was inverted in the *w*VulP genome. The structure of the nearby flanking regions was also conserved, bordered by IS elements and containing two identical hypothetical proteins and one ankyrin gene (97 to 99% amino acid sequence identity) (Figure 7). In *w*VulC and *w*VulM, this region was bordered by identical IS5 elements belonging to two different clusters (IS5_226 and IS5_519) (Supplementary Table S6).

## Discussion

Although studies on *Wolbachia* have gained momentum since the late 1990s, the mechanisms involved in host-*Wolbachia* interactions are still poorly understood (Landmann, 2019). Indeed, the search for bacterial factors responsible for the different phenotypes induced by *Wolbachia* has long been the subject of intensive study (Rice et al., 2017; Carpinone et al., 2018). The sequencing of numerous *Wolbachia* genomes over the last twenty years has provided access to all the bacterium’s genetic information and opened up the way to comparative genomic analyses (Kaur et al., 2021). The presence of prophages in most of these genomes was of particular interest, as these mobile elements could carry effectors of the feminizing phenotype of this endosymbiont, in particular genes involved in mutualistic relationships or in the manipulation of host reproduction (Bordenstein and Bordenstein, 2016). Although in most *Wolbachia* genomes the prophages appeared to be sedentary, it has been shown in the parasitoid wasp *Nasonia vitripennis* that they can still be active and propagate (Bordenstein et al., 2006). In this *Wolbachia* strain, it was also shown that the sedentarized copies had undergone numerous rearrangements (Bordenstein and Bordenstein, 2022). In the end, these prophage analyses identified the *cifA* and *cifB* genes as well as the *wmk* gene, involved in CI and MK, respectively (LePage et al., 2017; Perlmutter et al., 2019). While the genome of the *Wolbachia* strain of *Trichogramma* contained only degenerated prophage regions (Lindsey et al., 2016), two putative PI factors were identified in a degraded EAM (Fricke and Lindsey, 2024).

In this study, we performed a comparative genomic analysis of the three *Wolbachia* strains *w*VulC, *w*VulM, and *w*VulP infecting *A. vulgare*, focusing on the identification of putative feminizing factors.

Since their collection from the wild, the three *Wolbachia*-infected lineages of *A. vulgare* were stably maintained in the laboratory. The sex-ratio of the progenies as well as the presence of the corresponding *Wolbachia* strains in female genitors were regularly monitored by diagnostic PCR. Although multiple strains may coexist in *A. vulgare* populations, no individual has been found to be multiinfected (Verne et al., 2007). Moreover, feminization led to cytoplasmic sex determination in natural populations where all infected *Wolbachia*-infected females were ZZ reversed males (Juchault et al., 1993; Rigaud et al., 1997). This implies variations in offspring sex ratios as a function of *Wolbachia* transmission rate, as it has been demonstrated in *A. vulgare* populations infected with either *w*VulC or *w*VulM (Cordaux et al., 2004). In the three laboratory lineages of *A. vulgare*, male biased ratio may be thus observed in some offspring of infected females. Far from being surprising, this confirms that these females were necessarily ZZ inverted males, which can produce male biased offspring when the *Wolbachia* transmission rate falls below 50%. In contrast, the balanced male ratio observed in the uninfected control line was the result of chromosomal sex determination with individuals of ZZ and WZ genotypes. All these observations confirmed the feminizing phenotype of the three *Wolbachia* strains of *A. vulgare*, leading to transmission rate-dependent cytoplasmic sex determination.

Consistent with previous single gene (16S rDNA, *ftsZ*, *wsp*, and *GroE*) phylogenies (Bouchon et al., 1998; Cordaux et al., 2001; Wiwatanaratanabutr et al., 2009), as well as multi-locus sequence typing data (Sicard et al., 2014), *w*VulC, *w*VulM and *w*VulP belonged to the supergroup B forming a separated clade in phylogenomic analyses (bootstrap support 100, Figure 3). This reflected a general pattern of *Wolbachia* from terrestrial isopods, which clustered in a monophyletic clade (referred as the *Oni* clade for Oniscidea; (Cordaux et al., 2001)) within supergroup B. This result also confirmed the genetic proximity of the *w*VulC and *w*VulM strains.

The *w*VulC, *w*VulM, and *w*VulP genomes were between 1.7 and 1.6 Mb in size and contained between 1,700 and 1,500 genes, corresponding to a coding density of 75 to 67% (Table 1). These characteristics correlated with genome size, *w*VulC being the largest and *w*VulP the smallest. They were also consistent with the features of the B supergroup of *Wolbachia* which are known to have a larger number of genes than the A supergroup (Vancaester and Blaxter, 2023). The genome length of the three strains was among the longest known for *Wolbachia* (Scholz et al., 2020). These sizes were highly correlated with the extent of the mobilome (including prophages, IS elements and group II introns), representing a total of 415,545 bp for *w*VulC, 338,395 bp for *w*VulM and 287,918 bp for *w*VulP, i.e., from 24 to 18% of the genome. The high density of mobile and repeated elements in the three genomes, in particular IS belonging to the IS110 and IS256 families, could explain the genomic rearrangements observed (Figure 3; Supplementary Figures S2A–C). The copy number of group II introns was also one of the highest reported in *Wolbachia* genomes (Leclercq et al., 2011). These features were, to varying degrees, common to *Wolbachia* reproductive parasites, as demonstrated by the first sequenced genomes (Wu et al., 2004; Klasson et al., 2008, 2009). Particular attention was paid to the *w*VulC strain, as a Sanger sequence assembly (GCA_001027565.1) and *w*VulC inserts identified into the pill bug nuclear genome are publicly available (Leclercq et al., 2016). Consistently, genomic comparisons have confirmed that locally collinear blocks are conserved, but that significant genomic rearrangements are probably due to the impact of the mobilome, in particular the IS and group II introns (Figure 3; Supplementary Figures S2A–C, S3, S4). This is particularly obvious in the comparison with the nine scaffolds of the *f* element where, despite a high average nucleic identity, collinearity remains low. This pattern was also evident when only the longest scaffold of 2,798,100 bp (LYUU01002088.1) is included in the comparison (Leclercq et al., 2016). It has long been known that *Wolbachia* can recombine with several implications on the evolution of these bacteria (Jiggins et al., 2001; Werren and Bartos, 2001). Based on single gene analyses, two recombination events have been shown in feminizing *Wolbachia* and more specifically in *w*VulP (Verne et al., 2007) and complete pathway for homologous recombination pathway was found in *w*VulC (Badawi et al., 2014). *Wolbachia*’s great ability for recombination could therefore explain the genomic plasticity observed.

A complete biotin operon has been identified in the three genomes, suggesting that these *Wolbachia* strains were capable of synthesizing B vitamins. It was also conserved in the *f* element inserted into the genome of *A. vulgare*. Although generally absent from most *Wolbachia* genomes, a complete biotin operon has been identified in around fifteen *Wolbachia* strains (Beliavskaia et al., 2023). However, it has been shown that only the *Wolbachia w*Cle from the bedbug *Cimex lectularius* supplies B vitamins to its host (Nikoh et al., 2014). This beneficial symbiosis was not demonstrated in the other strains harboring the biotin operon. For example, biotin supplementation remains unlikely in the *w*CfeF strain of *Ctenochephalides felis*, since the *bioB* gene is frameshifted (Beliavskaia et al., 2023). Similarly, the *w*Oo strain of *Onchocerca ochengi* has a completely disrupted biotin operon (Darby et al., 2012; Nikoh et al., 2014). It therefore seems that this operon undergoes evolutionary events leading to its degradation, presumably due to the absence of a selective advantage for the host. In the particular case of *A. vulgare*, although the biotin operon is complete in the three *Wolbachia* genomes and also in the *f* element, and may supply B vitamins as well, the absence of an obligate association with the host may indicate that this contribution is not essential. Finally, the presence of IS and transposases in the flanking regions of the operon is consistent with an acquisition by lateral gene transfer, supported by the lack of congruence between the phylogenies of the biotin genes and the *Wolbachia* (Nikoh et al., 2014; Driscoll et al., 2020; Beliavskaia et al., 2023). Accordingly, phylogenetic analyses support the hypothesis of at least three independent acquisition of the biotin operon by Rickettsiales (Driscoll et al., 2020; Lefoulon et al., 2020).

Multiple copies of the WO prophages were identified in the genomes of all three feminizing strains. However, copy number differs from genome to genome, *w*VulC having 4 copies representing a total of 248,915 bp (including the EAM-like regions), followed by *w*VulM with three copies representing 182,635 bp and *w*VulP with only 2 copies representing 139,041 bp. This copy number contributes to the size of the genomes, with *w*VulC being the largest and *w*VulP the smallest, representing between 9 and 10% of the total. Multiple phage infections have been observed in many *Wolbachia* strains from different arthropod host species (Gavotte et al., 2006). In particular, PCR amplification of the minor capsid gene *orf7* showed the presence of 4 to 6 copies of the WO phage in the terrestrial isopods *A. vulgare*, *Porcellionides pruinosus*, and *Porcellio dilatatus* (Braquart-Varnier et al., 2005). In agreement with our genomic data, the four copies of the *orf7* amplified in *A. vulgare*, corresponded to the capsid assembly proteins (wVulc_000689, wVulc_000754, wVulc_001230, and wVulc_0001319), annotated, respectively, in the core regions of prophages WOVulC1_2, WOVulC3_4, WOVulC5_6, and WOVulC7_8 of the *w*VulC genome.

The prophage loci were not restricted to the core modules of the prophage regions, and WO-like Islands (Bordenstein and Bordenstein, 2022) corresponding to “relic” prophages have been annotated in all three genomes. Due to the presence of numerous hypothetical proteins, it was difficult to define the precise boundaries of these regions. However, a general pattern seemed to emerge for the three genomes with two main WO-like islands. The first island comprised two portal proteins associated with two ankyrin repeat proteins in *w*VulC (from wVulc_000522 to wVulm_000527) and in *w*VulM (from wVulm_000522 to wVulm_000527) and only one portal protein and one ANK gene in *w*VulP (from wVulp_000412 to wVulp_000415). The second WO-like island had a structure comprising a terminase large subunit, an ANK gene, a hypothetical protein, a DNA modification methylase, a Holliday junction resolvase and a RhuM domain containing protein. This module was colinear in all three genomes, ranging from wVulc_000813 to wVulc_000818 in *w*VulC, from wVulm_000751 to wVulm_000756 in *w*VulM, and, with reverse synteny, from wVulp_000974 to wVulp_000969 in *w*VulP. These results were in line with those observed in various *Wolbachia* strains where RhuM virulence genes are located close to prophage genes encoding Holliday junction resolvase and DNA methylase proteins (Kent and Bordenstein, 2010; Fallon, 2020). Finally, single prophage genes encoding proteins of head, baseplate and tail, flanked either by hypothetical proteins or mobile elements (group II intron and IS) were annotated in the three genomes. These regions are too short to constitute genomic islands, but they testify to WO prophage degradation processes in *Wolbachia* genomes (Bordenstein and Bordenstein, 2022). Apart from this common pattern, a small cluster of prophage WO-like genes was identified in *w*VulP. This region (wVulp_000778 to wVulp_000780), flanked by two hypothetical proteins, contained genes encoding one head-tail connector protein, one DUF3168 domain-containing protein and one phage tail tube protein.

The T4SS is an efficient way for *Wolbachia* to transfer DNA and/or proteins to eukaryotic cells (Lindsey, 2020). Indeed, putative T4SS substrates have been already described in *Wolbachia* (Sheehan et al., 2016; Rice et al., 2017; Carpinone et al., 2018). This secretion system, consisting of *virB* and *virD* genes clustered at two loci, was conserved in *Wolbachia* reproductive parasites (Pichon et al., 2009). In line with this work, the presence of these two operons were confirmed in the three genomes. The ubiquitous presence of these operons in *Wolbachia* strains strongly suggested that they were functional, and led to an extensive search for potential substrates (Rice et al., 2017; Carpinone et al., 2018). Off all the hundred proteins identified as putatively secreted by the T4SS, three were located in the prophage regions of all three strains and had homologs in the *f* element: one ankyrin repeat domain-containing protein, one HTH domain-containing protein and one hypothetical protein. ANK motifs in bacterial proteins were thought to mimic eukaryotic protein–protein interactions, enabling bacteria to interact with host factors (Jernigan and Bordenstein, 2014). T4SS-secreted ANK proteins that interact with host cells has already been identified in bacteria such as *Legionella pneumophila*, *Anaplasma phagocytophilum* or *Ehrlichia chaffeenis* (Rikihisa and Lin, 2010; Yu et al., 2018). They were therefore ideal candidates as symbiotic factors in reproductive parasitism. In addition, protein–protein interactions were demonstrated between an MK inducer, Oscar, a *Wolbachia* protein containing ankyrin repeats, and the Masc protein involved in both masculinization and dosage compensation in the moth *Ostrinia furnacalis* (Katsuma et al., 2022). The induced reduction in Masc accumulation led to the inhibition of masculinization and the failure of dosage compensation, resulting in the death of male offspring (Katsuma et al., 2022). Another MK factor (SpAID), a protein with ANK repeats and a OTU (ovarian tumor) deubiquitinase domain, has been identified in *Spiroplasma poulsonii* (Harumoto and Lemaitre, 2018). The authors proposed a model in which the OTU domain induced nuclear localization, allowing SpAID to interact through its ANK domain with the Male Specific Lethal complex, thereby disrupting dosage compensation that led to male-killing phenotype. Interestingly, this is the only ANK protein identified in the *Spiroplasma* genome. In *w*Mel genome, a MK gene candidate (*wmk*) encoding two HTH, XRE family DNA-binding domains has been identified in the EAM of the prophage WOMelB, next to the *cifA* and *cifB* genes that are involved in CI (Perlmutter et al., 2019). Homologs of *wmk* were also found in prophage EAMs from different MK strains. This protein likely interacts with DNA through its two HTH domains and could act as a transcriptional regulator potentially also targeting dosage compensation mechanisms. Based on this work, the ANK gene and the HTH-domain gene identified in this study were therefore be strong putative candidates for feminization. These genes were present, as expected, both in the feminizing *Wolbachia* genomes and in the *f* element, the latter also inducing a feminizing phenotype in the host into whose genome it has been inserted (Leclercq et al., 2016). In the three feminizing strains, the ANK gene was part of the WO prophage core modules whereas the HTH domain-containing gene was localized in an EAM, like the *Wolbachia* CI and MK factors (LePage et al., 2017; Shropshire et al., 2020). Nevertheless, putative factors inducing PI in parasitoid wasps have been identified in *Wolbachia* prophage relics (Fricke and Lindsey, 2024). This is why we also paid attention to a latrotoxin-related protein localized near isolated phage genes, evoking phage relics and present in all feminizing strains and the *f* element. After processing of the latrotoxin C-terminal domain, the toxin encoded by this gene may be able to form ion-permeable membrane pores leading to host cell lysis, as does the latrotoxin of widow spiders venom in the cells of their prey (Zhang et al., 2012). Spider latrotoxins appear to have been acquired by lateral transfer from a bacterial endosymbiont (Bordenstein and Bordenstein, 2016). Such toxins could also be involved in the feminizing phenotype. Indeed, previous data have shown that *Wolbachia* do not directly target the androgenic hormone neither its receptors, but more likely the nerve centres that control the activity of the receptors (Juchault and Legrand, 1985; Herran et al., 2021). The toxin could therefore target these nerve centers and disrupt androgen receptor function.

Overall, our study highlighted three strong candidates for feminization of *A. vulgare* males. Further experiments need to be performed to confirm that these candidates disrupt the androgenic hormone pathway. Indeed, these genes need to be expressed during embryonic development, at the stage where the androgenic hormone expression that induces male differentiation is inhibited in infected animals. Besides, the *Wolbachia* load increases just at this stage, defining the window of action enabling the bacteria to counteract the masculinizing effect of the androgen hormone and induce the development of male embryos into females (Herran et al., 2020). Monitoring the expression of these candidate genes during the development of the offspring of *Wolbachia*-infected females could provide further support for our hypotheses. To decipher the mechanisms of feminization, it will be necessary to identify host targets with which these candidates can interact.

## Data Availability

The *Wolbachia* genomes of *A. vulgare* recovered in this study were deposited in GenBank (https://www.ncbi.nlm.nih.gov/genbank) under BioProjects PRJNA1093130, PRJNA1093132, and PRJNA1093134 and the genomes are available under the accession numbers CP156068, CP156069, and CP156070.

## Footnotes

^1^ https://github.com/rrwick/Porechop

## Supplementary material

Supplementary Table S1 Sex ratio of progenies in four different laboratory lineages of *A. vulgare* over 5 years. BI, CP, and ZN were lineages naturally infected with *w*VulM, *w*VulP, and *w*VULC *Wolbachia* strains, respectively. BF was an uninfected lineage.

Supplementary Table S2 Pairwise comparison of average nucleotide identity (ANI) values between *w*VulC, *w*VulM, and *w*VulP genomes (*w*VulC as reference).

Supplementary Table S3 OrthoFinder analysis to identify orthologous groups of genes from the three *Wolbachia* genomes.

Supplementary Table S4 *Wolbachia* genomes used in the phylogenomic analysis.

Supplementary Table S5 Orthogroup analysis from 29 *Wolbachia* complete genomes.

Supplementary Table S6 Annotations of IS elements predicted by ISEScan analysis.

Supplementary Table S7 Annotations of group II intron-associated genes.

Supplementary Table S8 Annotations of the phage regions.

Supplementary Table S9 EffectiveDB predictions of Type IV Secretion Systems (T4SS) and potential effectors.

Supplementary Figure S1 Plot of mean male ratio of progeny from the four laboratory lines of *A. vulgare* (BF, BI, CP, and ZN) per year over five-years. BF lineage is uninfected whereas BI, CP, and ZN lineages are infected with *w*VulM, *w*VulP, and *w*VulC *Wolbachia* strains, respectively. White circles correspond to mean values and whiskers represent standard error.

Supplementary Figure S2 Alignment of the three *Wolbachia* genomes: **(A)** Collinearity between *w*VulC and *w*VulM. **(B)** Collinearity between *w*VulC and *w*VulP. **(C)** Collinearity between *w*VulM and *w*VulP. Pairwise comparisons were performed by BLASTn using FastANI 1.3 software. Each red line segment indicates a reciprocal match between two sequences. The colored bar indicates the percentage of identity. Homologous regions are indicated by segments of the same color.

Supplementary Figure S3 Alignment of the complete *w*VulC genome obtained by ONT sequencing technology (this study) with the previous draft genome (GCA_001027565.1; unpublished) obtained by Sanger technology. Collinearity detected by BLASTn using FastANI 1.3 software. Each red line segment denotes a reciprocal match between two sequences. Homologous regions are indicated by segments of the same color. The colored bar indicates the percentage of identity.

Supplementary Figure S4 Alignment of the complete *w*VulC genome with the concatenated eight scaffolds of the *Wolbachia* inserts (*f* element; Leclercq et al., 2016) in the nuclear genome of *A. vulgare*. Collinearity detected by BLASTn using FastANI 1.3 software. Each red line segment denotes a reciprocal match between two sequences. Homologous regions are indicated by segments of the same color. The colored bar indicates the percentage of identity.
