# Supplementary material for: Three feminizing Wolbachia strains in a single host species: comparative genomics paves the way for identifying sex reversal factors
Source: Front Microbiol. 2024 Aug 22;15:1416057. doi: 10.3389/fmicb.2024.1416057 (PMC11376236; doi:10.3389/fmicb.2024.1416057)
Supplement: Supplementary Table S2 — Pairwise comparison of average nucleotide identity (ANI) values between wVulC, wVulM, and wVulP genomes (wVulC as reference). [file Table_2.DOCX]

**Supplementary Table S2.** Pairwise comparison of average nucleotide identity (ANI) values between *w*VulC, *w*VulM and *w*VulP genomes (*w*VulC as reference).

|  | ANI value (%) | Count of Bidirectional Fragment Mappings | Total Query Fragments |
| --- | --- | --- | --- |
| wVulM | 99.4158 | 541 | 546 |
| wVulP | 98.6474 | 473 | 522 |
