# Supplementary material for: Three feminizing Wolbachia strains in a single host species: comparative genomics paves the way for identifying sex reversal factors
Source: Front Microbiol. 2024 Aug 22;15:1416057. doi: 10.3389/fmicb.2024.1416057 (PMC11376236; doi:10.3389/fmicb.2024.1416057)
Supplement: Supplementary Table S4 — Wolbachia genomes used in the phylogenomic analysis. [file Table_4.DOCX]

**Supplementary Table S4.** *Wolbachia* genomes used in the phylogenomic analysis

| Strain | Supergroup | Host taxon | Host | Genome size (Mb) | Accession number | Reference |
| --- | --- | --- | --- | --- | --- | --- |
| *w*Mel | A | Diptera | *Drosophila melanogaster* | 1.268 | CP046925 | Wu et al., 2004 |
| *w*Ri | A | Diptera | *Drosophila simulans* | 1.446 | CP001391 | Klasson et al., 2009 |
| *w*Ha | A | Diptera | *Drosophila simulans* | 1.296 | CP003884 | Ellegaard et al., 2013 |
| *w*Au | A | Diptera | *Drosophila simulans* | 1.268 | LK055284 | Sutton et al., 2014 |
| *w*Cin2USA1 | A | Diptera | *Rhagoletis cingulata* | 1.538 | CP072012 | Wolfe et al., unpublished |
| *w*Pip | B | Diptera | *Culex quinquefasciatus* Pel | 1.482 | AM999887 | Klasson et al., 2008 |
| *w*Mau | B | Diptera | *Drosophila mauritiana* | 1.274 | CP034335 | Lefoulon et al., 2019 |
| *w*No | B | Diptera | *Drosophila simulans* | 1.302 | CP003883 | Ellegaard et al., 2013 |
| *w*Meg | B | Diptera | *Chrysomya megacephala* | 1.377 | CP021120 | Junqueira, unpublished |
| *w*Di | B | Hemiptera | *Diaphorina citri* | 1.656 | CP051608 | Petrone et al., unpublished |
| *w*AlbB | B | Diptera | *Aedes albopictus* | 1.484 | CP031221 | Sinha et al., 2019 |
| *w*BtaB | B | Hemiptera | *Bemisia tabaci* | 1.306 | CP016430 | Zhu et al., unpublished |
| *w*Ccep | B | Lepidoptera | *Corcyra cephalonica* | 1.359 | CP087954 | Li and Li, unpublished |
| *w*AnD | B | Diptera | *Anopheles demeilloni* | 1.231 | CP084694 | Walker et al., 2021 |
| *w*Tpre | B | Hymenoptera | *Trichogramma pretiosum* | 1.133 | CM003641 | Lindsey et al., unpublished |
| *w*Spic | B | Lepidoptera | *Spodoptera picta* | 1.339 | CP067976 | Niu, unpublished |
| *w*Fur | B | Lepidoptera | *Ostrinia furnacalis* | 1.321 | CP096925 | Muro and Katsuma, unpublished |
| *w*Sca | B | Lepidoptera | *Ostrinia scapulalis* | 1.320 | CP096926 | Muro and Katsuma, unpublished |
| *w*Dimm | C | Nematode | *Dirofilaria immitis* | 0.920 | NZ_CP046578 | Lefoulon et al., 2020 |
| *w*Oo | C | Nematode | *Onchocerca ochengi* | 0.958 | HE660029 | Darby et al., 2012 |
| *w*Ov | C | Nematode | *Onchocerca volvulus* | 0.961 | NZ_HG810405 | Cotton et al., 2016 |
| *w*Lsig | D | Nematode | *Litomosoides sigmodontis* | 1.046 | CP046577 | Lefoulon et al., 2020 |
| *w*Wb | D | Nematode | *Wuchereria bancrofti* | 1.268 | GCA_002204235.2 | Chung et al., 2017 |
| *w*Bm | D | Nematode | *Brugia malayi* | 1.080 | AE017321 | Foster et al., 2005 |
| *w*Fol | E | Collembola | *Folsomia candida* | 1.802 | CP015510 | Faddeeva-Vakhrusheva et al., 2017 |
| *w*Cle | F | Hemiptera | *Cimex lectularius* | 1.250 | AP013028 | Nikoh et al., 2014 |

**References**

Chung, M., Small, S. T., Serre, D., Zimmerman, P. A., & Dunning Hotopp, J. C. (2017). Draft genome sequence of the Wolbachia endosymbiont of Wuchereria bancrofti wWb. *Pathogens and Disease*, *75*(9), ftx115. https://doi.org/10.1093/femspd/ftx115

Cotton, J. A., Bennuru, S., Grote, A., Harsha, B., Tracey, A., Beech, R., Doyle, S. R., Dunn, M., Hotopp, J. C. D., Holroyd, N., Kikuchi, T., Lambert, O., Mhashilkar, A., Mutowo, P., Nursimulu, N., Ribeiro, J. M. C., Rogers, M. B., Stanley, E., Swapna, L. S., … Lustigman, S. (2016). The genome of Onchocerca volvulus, agent of river blindness. *Nature Microbiology*, *2*(2), 1‑12. https://doi.org/10.1038/nmicrobiol.2016.216

Darby, A. C., Armstrong, S. D., Bah, G. S., Kaur, G., Hughes, M. A., Kay, S. M., Koldkjær, P., Rainbow, L., Radford, A. D., Blaxter, M. L., Tanya, V. N., Trees, A. J., Cordaux, R., Wastling, J. M., & Makepeace, B. L. (2012). Analysis of gene expression from the Wolbachia genome of a filarial nematode supports both metabolic and defensive roles within the symbiosis. *Genome Research*, *22*(12), 2467‑2477. https://doi.org/10.1101/gr.138420.112

Ellegaard, K. M., Klasson, L., Näslund, K., Bourtzis, K., & Andersson, S. G. E. (2013). Comparative genomics of Wolbachia and the bacterial species concept. *PLoS Genetics*, *9*(4), e1003381. https://doi.org/10.1371/journal.pgen.1003381

Faddeeva-Vakhrusheva, A., Kraaijeveld, K., Derks, M. F. L., Anvar, S. Y., Agamennone, V., Suring, W., Kampfraath, A. A., Ellers, J., Le Ngoc, G., van Gestel, C. A. M., Mariën, J., Smit, S., van Straalen, N. M., & Roelofs, D. (2017). Coping with living in the soil : The genome of the parthenogenetic springtail Folsomia candida. *BMC Genomics*, *18*(1), 493. https://doi.org/10.1186/s12864-017-3852-x

Foster, J., Ganatra, M., Kamal, I., Ware, J., Makarova, K., Ivanova, N., Bhattacharyya, A., Kapatral, V., Kumar, S., Posfai, J., Vincze, T., Ingram, J., Moran, L., Lapidus, A., Omelchenko, M., Kyrpides, N., Ghedin, E., Wang, S., Goltsman, E., … Slatko, B. (2005). The Wolbachia genome of Brugia malayi : Endosymbiont evolution within a human pathogenic nematode. *PLoS Biology*, *3*(4), e121. https://doi.org/10.1371/journal.pbio.0030121

Klasson, L., Walker, T., Sebaihia, M., Sanders, M. J., Quail, M. A., Lord, A., Sanders, S., Earl, J., O’Neill, S. L., Thomson, N., Sinkins, S. P., & Parkhill, J. (2008). Genome evolution of Wolbachia strain wPip from the Culex pipiens group. *Molecular Biology and Evolution*, *25*(9), 1877‑1887. https://doi.org/10.1093/molbev/msn133

Klasson, L., Westberg, J., Sapountzis, P., Näslund, K., Lutnaes, Y., Darby, A. C., Veneti, Z., Chen, L., Braig, H. R., Garrett, R., Bourtzis, K., & Andersson, S. G. E. (2009). The mosaic genome structure of the Wolbachia wRi strain infecting Drosophila simulans. *Proceedings of the National Academy of Sciences of the United States of America*, *106*(14), 5725‑5730. https://doi.org/10.1073/pnas.0810753106

Lefoulon, E., Clark, T., Guerrero, R., Cañizales, I., Cardenas-Callirgos, J. M., Junker, K., Vallarino-Lhermitte, N., Makepeace, B. L., Darby, A. C., Foster, J. M., Martin, C., & Slatko, B. E. (2020). Diminutive, degraded but dissimilar : Wolbachia genomes from filarial nematodes do not conform to a single paradigm. *Microbial Genomics*, *6*(12), mgen000487. https://doi.org/10.1099/mgen.0.000487

Lefoulon, E., Vaisman, N., Frydman, H. M., Sun, L., Voland, L., Foster, J. M., & Slatko, B. E. (2019). Large Enriched Fragment Targeted Sequencing (LEFT-SEQ) Applied to Capture of Wolbachia Genomes. *Scientific Reports*, *9*(1), 5939. https://doi.org/10.1038/s41598-019-42454-w

Nikoh, N., Hosokawa, T., Moriyama, M., Oshima, K., Hattori, M., & Fukatsu, T. (2014). Evolutionary origin of insect-Wolbachia nutritional mutualism. *Proceedings of the National Academy of Sciences of the United States of America*, *111*(28), 10257‑10262. https://doi.org/10.1073/pnas.1409284111

Sinha A, Li Z, Sun L, Carlow CKS. Complete Genome Sequence of the *Wolbachia* wAlbB Endosymbiont of *Aedes albopictus*. *Genome Biol Evol.* 2019 11(3):706-720. doi: 10.1093/gbe/evz025.

Sutton, E. R., Harris, S. R., Parkhill, J., & Sinkins, S. P. (2014). Comparative genome analysis of Wolbachia strain wAu. *BMC Genomics*, *15*(1), 928. https://doi.org/10.1186/1471-2164-15-928

Walker,T., Quek,S., Jeffries,C.L., Bandibabone,J., Dhokiya,V., Bamou,R., Kristan,M., Messenger,L.A., Gidley,A., Hornett,E.A., Anderson,E.R., Cansado-Utrilla,C., Hegde,S., Bantuzeko,C., Stevenson,J.C., Lobo,N.F., Wagstaff,S.C., Nkondjio,C.A., Irish,S.R., Heinz,E. and Hughes,G.L. (2021) Stable high-density and maternally inherited *Wolbachia* infections in *Anopheles* *moucheti* and *Anopheles* *demeilloni* mosquitoes. *Curr Biol* 31 (11), 2310-2320. doi: 10.1016/j.cub.2021.03.056.

Wu, M., Sun, L. V., Vamathevan, J., Riegler, M., Deboy, R., Brownlie, J. C., McGraw, E. A., Martin, W., Esser, C., Ahmadinejad, N., Wiegand, C., Madupu, R., Beanan, M. J., Brinkac, L. M., Daugherty, S. C., Durkin, A. S., Kolonay, J. F., Nelson, W. C., Mohamoud, Y., … Eisen, J. A. (2004). Phylogenomics of the Reproductive Parasite Wolbachia pipientis wMel : A Streamlined Genome Overrun by Mobile Genetic Elements. *PLOS Biology*, *2*(3), e69. https://doi.org/10.1371/journal.pbio.0020069
