# Supplementary figures and images for: Three feminizing Wolbachia strains in a single host species: comparative genomics paves the way for identifying sex reversal factors
Source: Front Microbiol. 2024 Aug 22;15:1416057. doi: 10.3389/fmicb.2024.1416057 (PMC11376236; doi:10.3389/fmicb.2024.1416057)

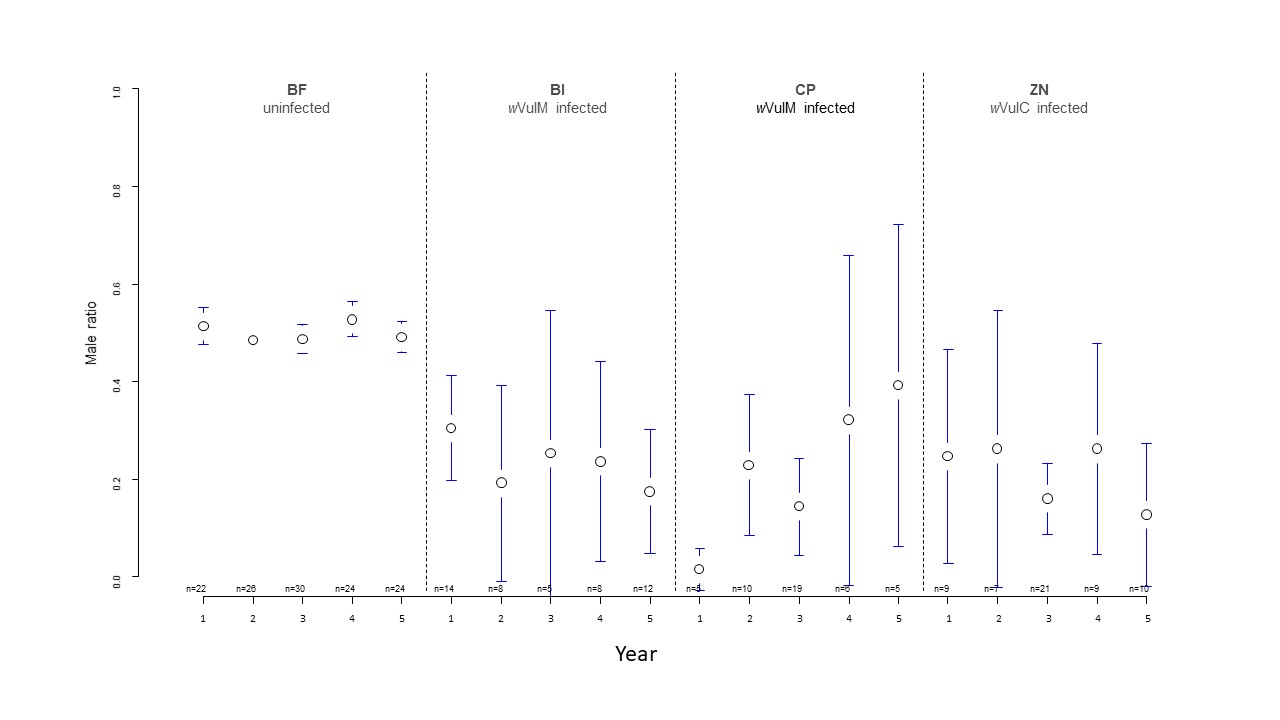

Supplement: Supplementary Figure S1 — Plot of mean male ratio of progeny from the four laboratory lines of A. vulgare (BF, BI, CP, and ZN) per year over five-years. BF lineage is uninfected whereas BI, CP, and ZN lineages are infected with wVulM, wVulP, and wVulC Wolbachia strains, respectively. White circles correspond to mean values and whiskers represent standard error. [file Image_1.JPEG]

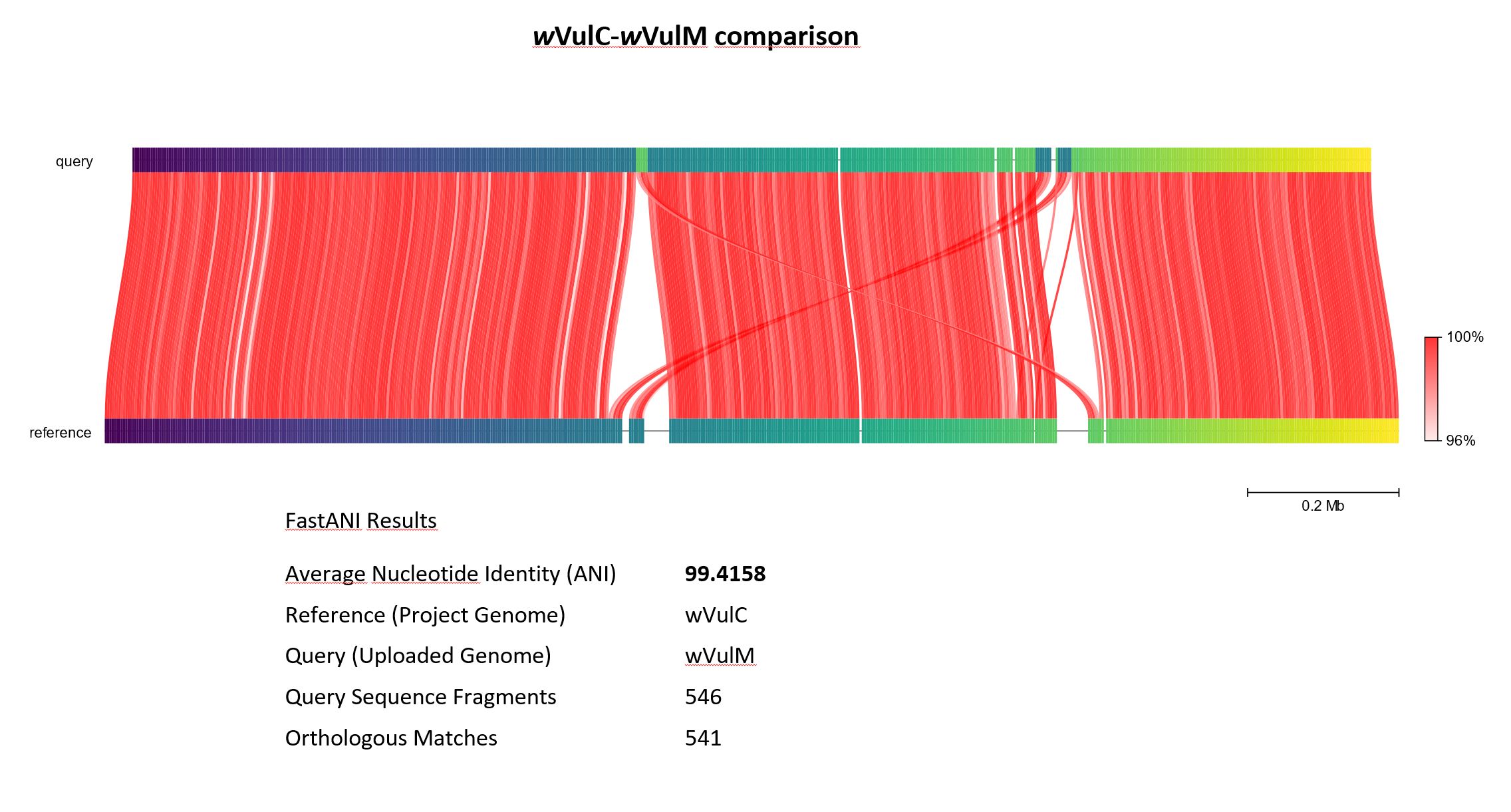

Supplement: Supplementary Figure S2 — Alignment of the three Wolbachia genomes: (A) Collinearity between wVulC and wVulM. (B) Collinearity between wVulC and wVulP. (C) Collinearity between wVulM and wVulP. Pairwise comparisons were performed by BLASTn using FastANI 1.3 software. Each red line segment indicates a reciprocal match between two sequences. The colored bar indicates the percentage of identity. Homologous regions are indicated by segments of the same color. [file Image_2.JPEG]

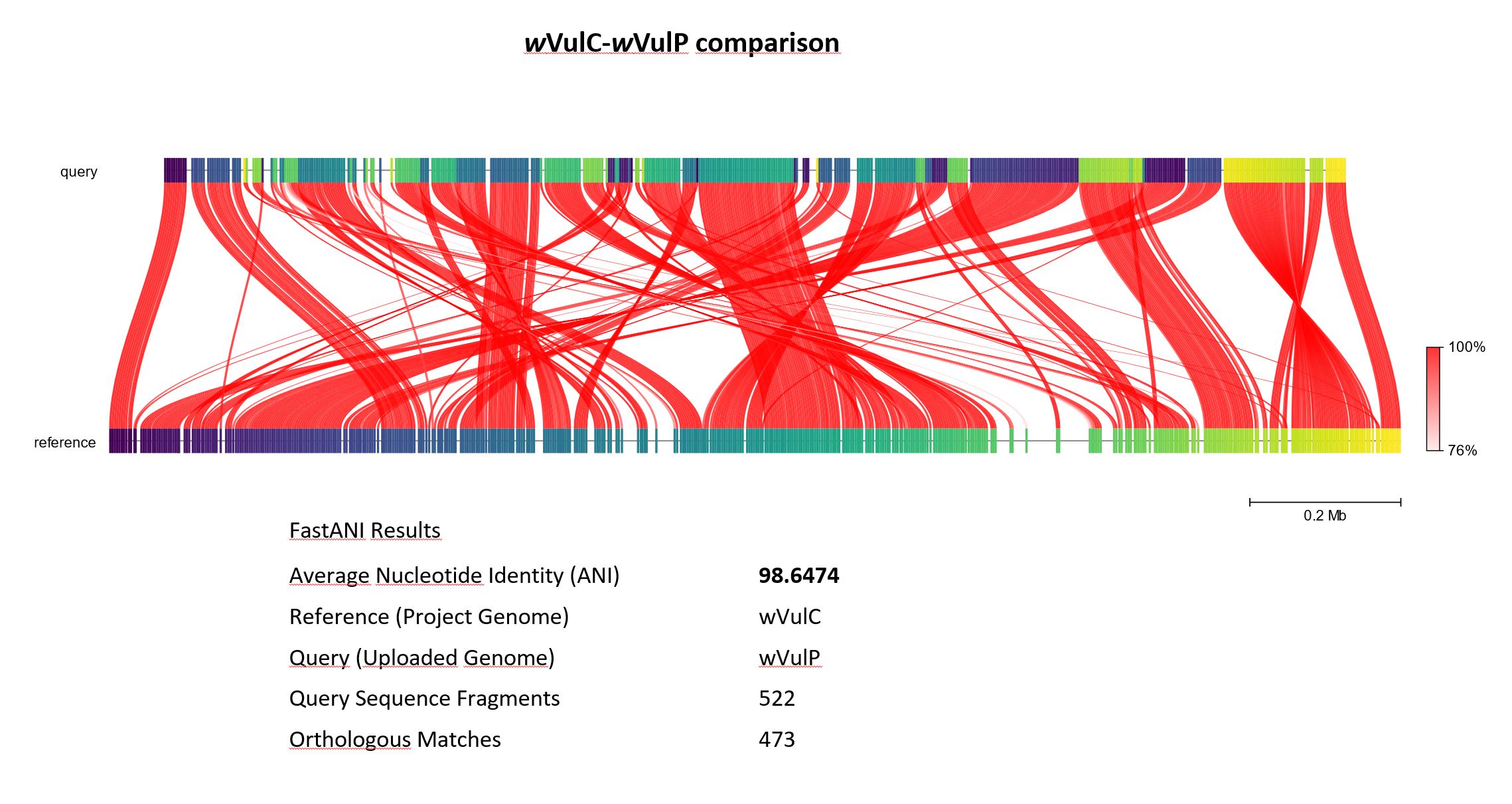

Supplement: Supplementary Figure S3 — Alignment of the complete wVulC genome obtained by ONT sequencing technology (this study) with the previous draft genome (GCA_001027565.1; unpublished) obtained by Sanger technology. Collinearity detected by BLASTn using FastANI 1.3 software. Each red line segment denotes a reciprocal match between two sequences. Homologous regions are indicated by segments of the same color. The colored bar indicates the percentage of identity. [file Image_3.JPEG]

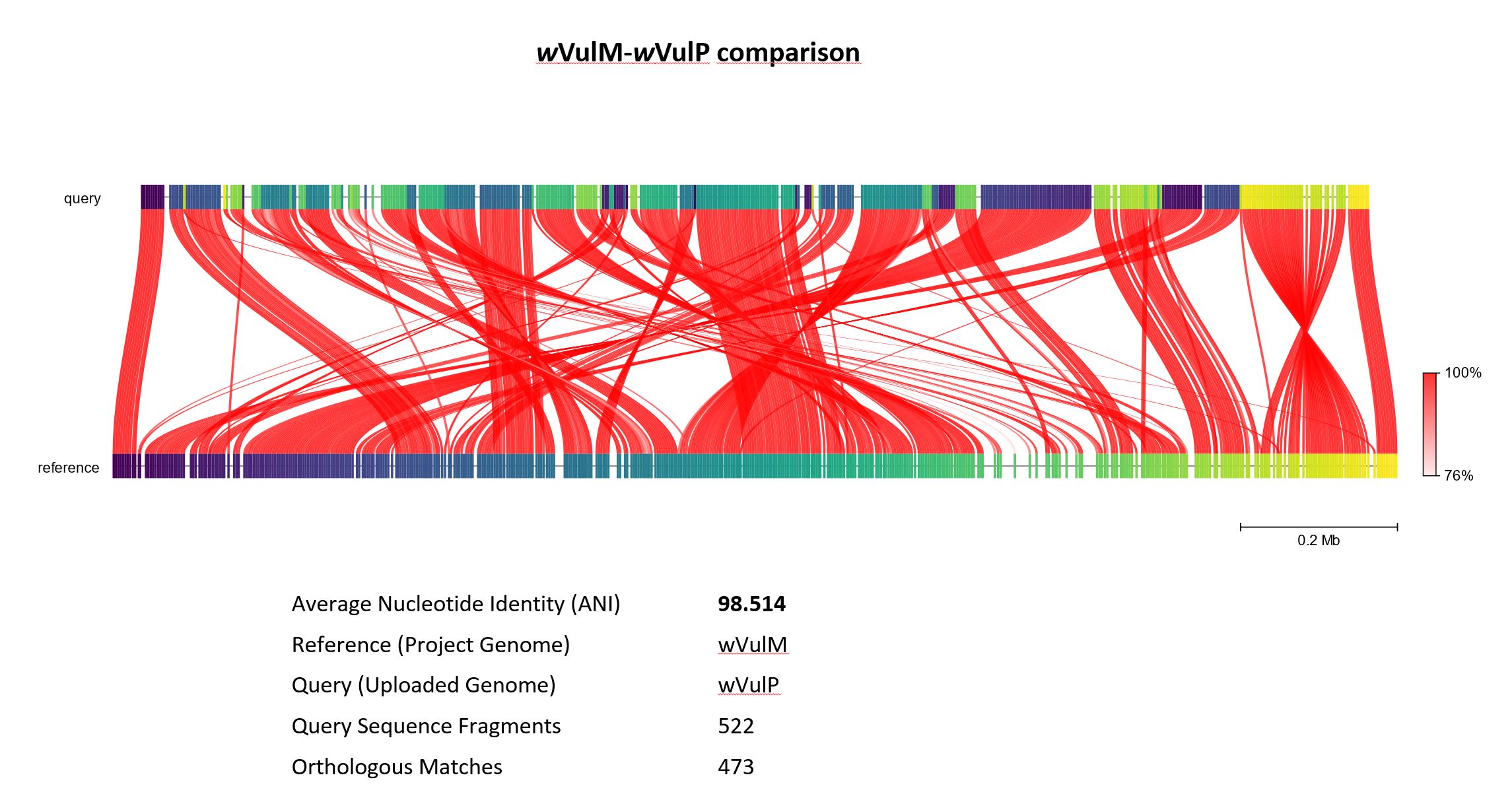

Supplement: Supplementary Figure S4 — Alignment of the complete wVulC genome with the concatenated eight scaffolds of the Wolbachia inserts (f element; Leclercq et al., 2016) in the nuclear genome of A. vulgare. Collinearity detected by BLASTn using FastANI 1.3 software. Each red line segment denotes a reciprocal match between two sequences. Homologous regions are indicated by segments of the same color. The colored bar indicates the percentage of identity. [file Image_4.JPEG]

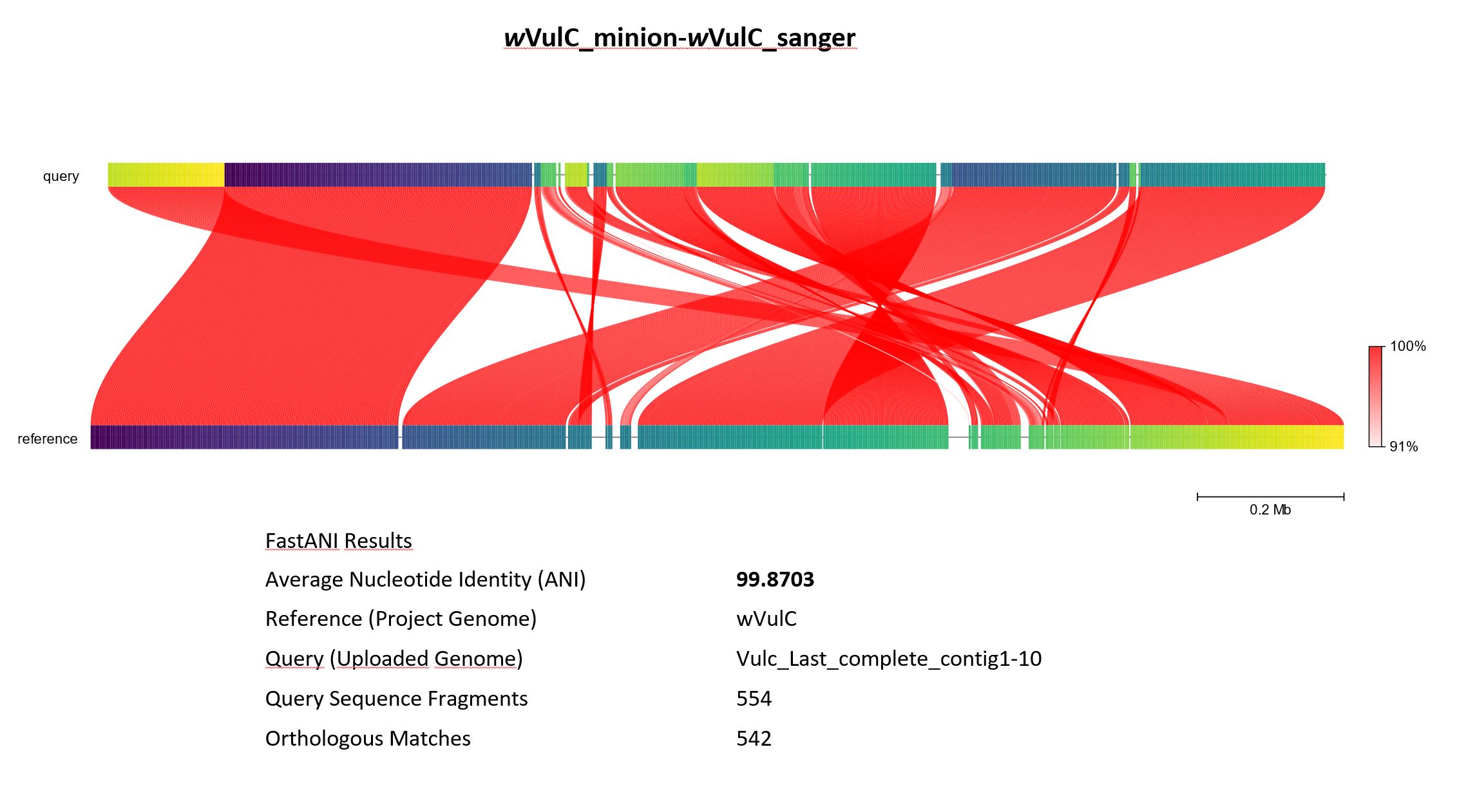

Supplement: Supplementary file 14 [file Image_5.JPEG]

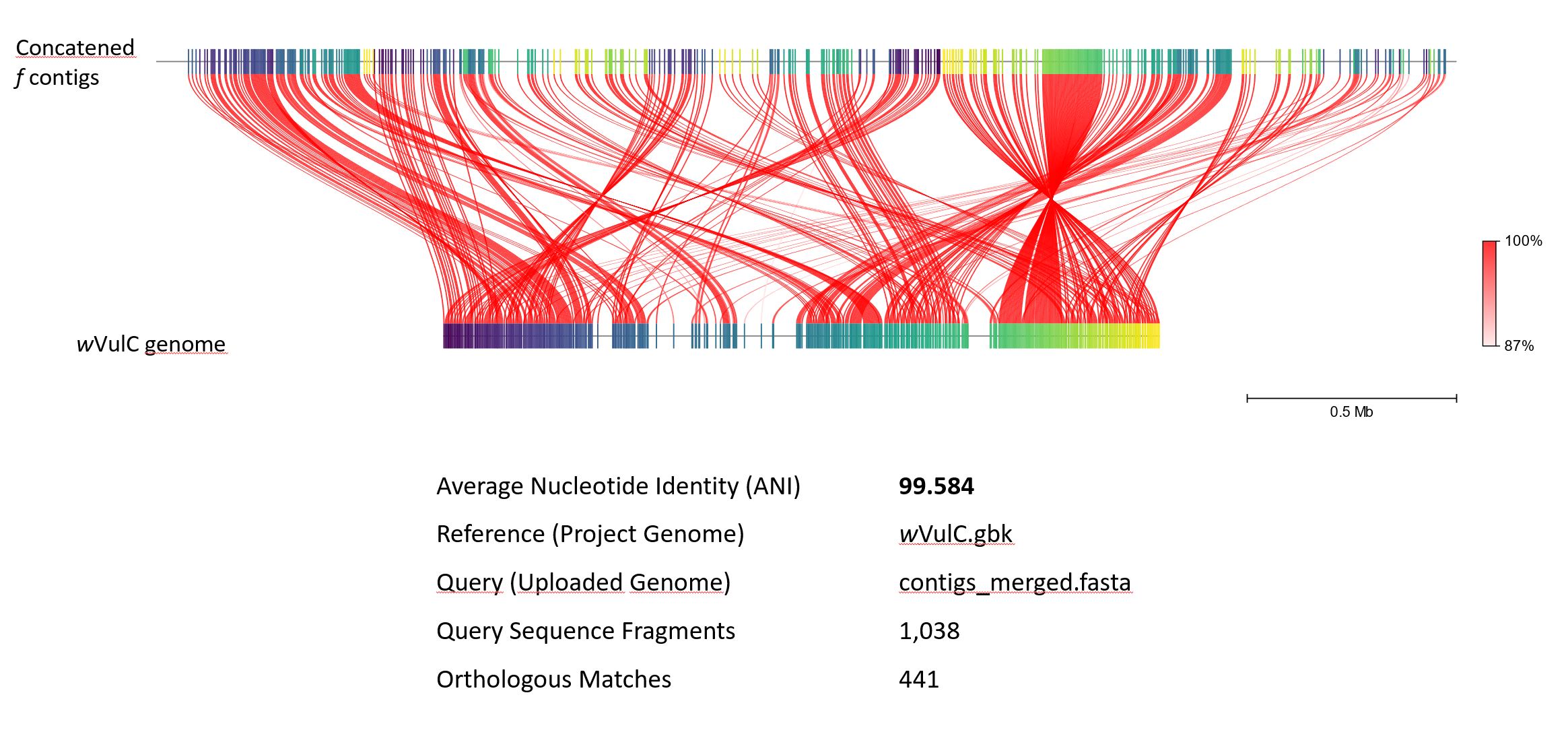

Supplement: Supplementary file 15 [file Image_6.JPEG]
